# Immune cellular patterns of distribution affect outcomes of patients with non-small cell lung cancer

**DOI:** 10.1038/s41467-023-37905-y

**Published:** 2023-04-25

**Authors:** Edwin Roger Parra, Jiexin Zhang, Mei Jiang, Auriole Tamegnon, Renganayaki Krishna Pandurengan, Carmen Behrens, Luisa Solis, Cara Haymaker, John Victor Heymach, Cesar Moran, Jack J. Lee, Don Gibbons, Ignacio Ivan Wistuba

**Affiliations:** 1 Departments of Translational Molecular Pathology, The University of Texas MD Anderson Cancer Center, Houston, TX USA; 2 Departments of Bioinformatics and Computational Biology, The University of Texas MD Anderson Cancer Center, Houston, TX USA; 3 Departments of Thoracic/Head and Neck Medical Oncology, The University of Texas MD Anderson Cancer Center, Houston, TX USA; 4 Departments of Pathology, The University of Texas MD Anderson Cancer Center, Houston, TX USA; 5 Departments of Biostatistics, The University of Texas MD Anderson Cancer Center, Houston, TX USA; 6 Departments of Molecular and Cellular Oncology, The University of Texas MD Anderson Cancer Center, Houston, TX USA

**Keywords:** Cancer microenvironment, Non-small-cell lung cancer, Non-small-cell lung cancer

## Abstract

Studying the cellular geographic distribution in non-small cell lung cancer is essential to understand the roles of cell populations in this type of tumor. In this study, we characterize the spatial cellular distribution of immune cell populations using 23 makers placed in five multiplex immunofluorescence panels and their associations with clinicopathologic variables and outcomes. Our results demonstrate two cellular distribution patterns—an unmixed pattern mostly related to immunoprotective cells and a mixed pattern mostly related to immunosuppressive cells. Distance analysis shows that T-cells expressing immune checkpoints are closer to malignant cells than other cells. Combining the cellular distribution patterns with cellular distances, we can identify four groups related to inflamed and not-inflamed tumors. Cellular distribution patterns and distance are associated with survival in univariate and multivariable analyses. Spatial distribution is a tool to better understand the tumor microenvironment, predict outcomes, and may can help select therapeutic interventions.

## Abstract (web summary)

The spatial distribution of cellular compartments within the tumour microenvironment in non-small cell lung cancer (NSCLC) remains to be investigated. Here, the authors identify distinct cell populations of tumour cells and tumour-associated immune cell phenotypes with different spatial distributions in NSCLC.

## Introduction

Despite recent advances in chemotherapy and immunotherapy, lung cancer, particularly non-small cell lung cancer (NSCLC), remains one of the most commonly diagnosed malignancies and often has poor overall outcomes^1^. Several analyses of NSCLC patients showed that adjuvant chemotherapy improved 5-year overall survival (OS) rates by only 5.4%^2^. Another meta-analysis of 1154 patients with stage II-III NSCLC showed that neoadjuvant chemotherapy with surgery was superior to surgery alone but had no benefit compared with adjuvant chemotherapy^3^. Moreover, the clinical effects of adjuvant tyrosine kinase inhibitors or anaplastic lymphoma kinase inhibitors in NSCLC remain limited^4^. Antibodies targeting immune checkpoints^5^ in NSCLC were recently shown to have a survival benefit^6,7^, improving 5-year OS rates in 20% of unselected patients and up to 40% of patients with high PD-L1 expression^8^. However, despite these promising results, a substantial proportion of patients receiving these treatments exhibited disease progression^9^.

Studying the interaction between malignant cells and tumor-associated immune cells (TAICs) using spatial distribution is essential to identify possible factors of tumor progression, relapse, or outcomes. This has been demonstrated not only in NSCLC^10^ but also in other tumor types, such as breast cancer^11^ and colon cancer^12^. Malignant cells can utilize various pathways to avoid immune surveillance^13,14^, and identifying such mechanisms of progression can help identify potential new targeting strategies for lung cancer immunotherapy^15^. Spatial mapping characterization of the immune can be related to prognostic indicators^16^, associated with the dysfunctional signature observed in melanoma tumors^17^, or interfere with other immune cells’ activation, maturation, and intratumoral distribution^18^. In addition, immune cell regulation^19^ and distribution can facilitate other cell inhibitors’ action in the tumor^20^, including a pro-tumorigenic microenvironment that can resist treatment^21^. On the other hand, the proximity of cytotoxic T-cells to tumor cells^22^ and the densities of those cells can benefit outcomes^23,24^.

The current study aimed to characterize the cellular composition of NSCLC and examine the spatial distribution of cell populations in NSCLC using multiplex immunofluorescence (mIF) panels. We also analyzed associations between cellular spatial distribution and clinicopathologic features and molecular profiles of NSCLC.

In the current study, we perform tumor immunoprofiling using 23 markers placed in five mIF panels staining on a cohort of NSCLC. Overall, we identify two patterns of cellular distribution—mixed and unmixed—related to T-cells, B-cells, macrophages, and PMNs as the primary cell phenotypes. Immune cellular distribution patterns and distance between malignant cells and various cell phenotypes show different associations with clinicopathologic characteristics, including smoking status, tumor size, final tumor stage, and mutational status. We further leverage Kaplan–Meier survival curves and Cox proportional hazards models showing that densities, distribution patterns, and distances from malignant cells to different cell phenotypes are also associated with outcomes. Finally, we can identify four groups of cellular immunologic patterns associated with cellular phenotype densities and outcomes according to the Cox proportional hazards regression model.

## Results

**Fig. 1:**
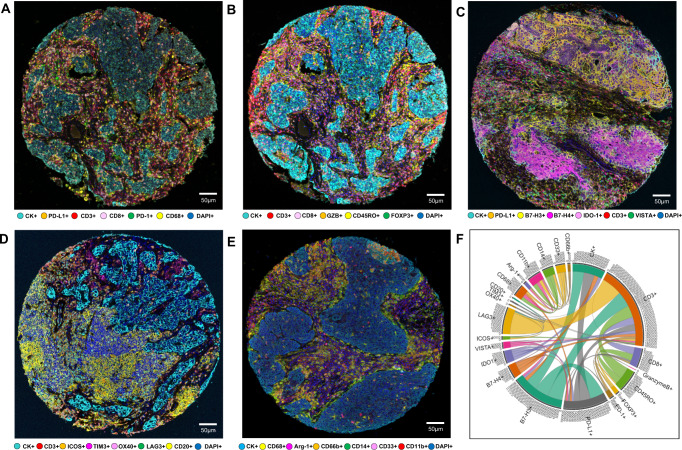
Representative examples of multispectral images from non-small cell lung cancer tissue microarray specimens, with their chord diagram of the markers. Composite spectral mixing images from multiplex immunofluorescence (mIF; 20× magnification, scale bars represent 50 µm on each image) is shown for (**A**) panel 1: cytokeratin (CK), CD3, CD8, PD-1, PD-L1, and CD68; (**B**) panel 2: CK, CD3, CD8, CD45RO, granzyme B (GZB), and FOXP3; (**C**) panel 3: CK, CD3, PD-L1, B7-H3, B7-H4, IDO-1; and VISTA; (**D**) panel 4: CK, CD3, ICOS, LAG3, OX40, TIM3, and CD20; (**E**) panel 5: CK, Arg-1, CD11b, CD14, CD33, CD66b, and CD68. **F** Chord diagram visualization showing the diversity of inter-relationships between markers’ co-expression, including all the markers in the five mIF panels. Data from 225 samples was used. Experiments and quantifications related to the presented results were conducted once. The images were generated using Vectra-Polaris 1.0.13 scanner system and InForm 2.4.8 image analysis software (Akoya Biosciences). The chord diagram was generated using all tumor cores from all mIF panels by R studio software version 3.6.1. (Source data is provided as a source data file).

**Fig. 2:**
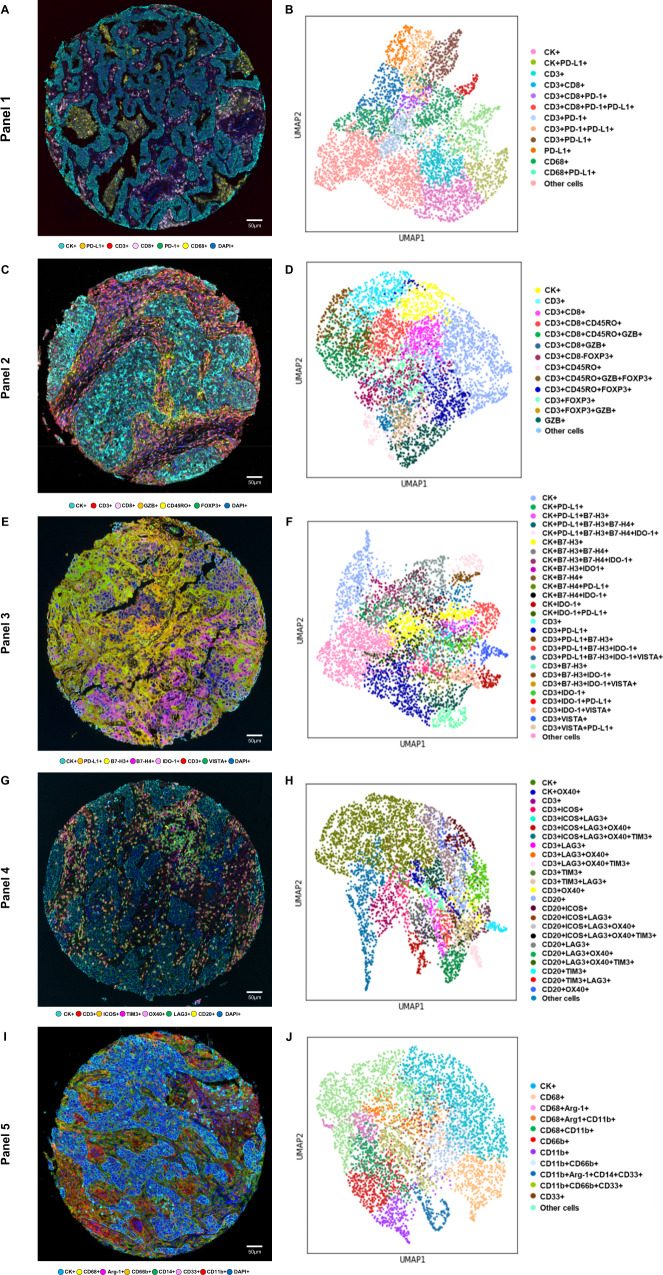
Representative examples of multispectral images and uniform manifold approximation and projection (UMAP) to identify cell types from non-small cell lung cancer tissue. Composite spectral mixing images from multiplex immunofluorescence (mIF; 20× magnification, scale bars represent 50 µm on each image) showing colored marker co-expression for (**A**) panel 1, (**C**) panel 2, (**E**) panel 3, (**G**) panel 4, and (**I**) panel 5. Color-coded UMAP visualizations show cell types identified by mIF panels: (**B**) 13 major cell types identified in panel 1, (**D**) 14 major cell types identified in panel 2, (**F**) 27 major cell types identified in panel 3, (**H**) 25 major cell types identified in panel 4, and (**J**) 12 major cell types identified in panel 5. Data from 225 samples was used. Experiments and quantifications related to the presented results were conducted once. mIF images were generated using Vectra-Polaris 1.0.13 scanner system and InForm 2.4.8 image analysis software (Akoya Biosciences), and UMAP visualizations were generated using the markers from each mIF panel by Python v.3.8.9. (Source data is provided as a source data file).

We analyzed expression of 23 markers, including CK, CD3, CD8, CD68, GZB, CD45RO, FOXP3, PD-1, PD-L1, B7-H3, B7-H4, IDO-1, VISTA, ICOS, LAG3, OX40, TIM3, CD20, Arg-1, CD11b, CD14, CD66b, and CD33, placed in five mIF panels. We identified different cell phenotypes by marker co-expression, as shown in Fig. 1. Cord plot visualization helped to show the inter-relationships between markers and the co-expression of the markers together as well as by individual panel (Fig. 1, Supplementary Fig. 1). Dimension reduction plots were used to help visualize and identify the different cell phenotypes detected in each mIF panel based on marker co-expression (Fig. 2).

### Co-expression of immune checkpoint molecules on malignant cells

The inter-relationships between markers through cord plots and UMAP showed that multiple immune checkpoints are expressed simultaneously by malignant cells and TAICs. We found that PD-L1, B7-H3, B7-H4, and IDO-1 immune checkpoints were expressed by malignant cells, in various densities (Fig. 2, Supplementary Fig. 1). In addition, our dataset captured some other cell populations less frequently observed, such as malignant cells expressing OX40 and other combinations of immune checkpoints (Fig. 2), which would be expected to escape from the phenotypes listed in Supplementary Table 1. A total of 14 malignant cell phenotypes were observed using panel 2. This illustrates the heterogeneity of marker co-expression, suggesting that several pathways may be activated in the malignant cells as part of their escape from immune surveillance. Overall, the cellular densities of the most frequently observed checkpoint molecules were higher in malignant cells from SCC than in those from ADC. The most predominant immune checkpoint expressed in malignant cells from ADC and SCC was B7-H3 (median, 307.79 cells/mm^2^), followed by PD-L1 (median, 77.265 cells/mm^2^), OX40 (median 25.38 cells/mm^2^), B7-H4 (median, 19.17 cells/mm^2^), and IDO-1 (median, 7.08 cells/mm^2^), and significantly higher densities of B7-H3 and B7-H4 were observed in SCC than in ADC (*P* < 0.001 for both immune checkpoints), whereas significantly higher densities of IDO-1 were observed in ADC than in SCC (*P* = 0.015; Supplementary Table 2).

### Characterization of T-cell and B-cell populations

The most common T-cell and B-cell subpopulation densities are shown in Supplementary Table 2 and Supplementary Fig. 1. Although we observed high amounts of classic T-cells and B-cells, such as CD3 + CD8 + cytotoxic T-cells (median, 136.51 cells/mm^2^), CD3 + CD45RO + memory T-cells (median, 57.39 cells/mm^2^), CD3 + CD8 + CD45RO + cytotoxic memory T-cells (median, 25.44 cells/mm^2^), and CD20 + B-cells (median, 80.07 cells/mm^2^), we also observed substantial densities of suppressive T-cells such as CD3 + CD8^neg^FOXP3 + regulatory T-cells (median, 24.10 cells/mm^2^), as well as T-cells and B-cells expressing other suppressive markers. Overall, we observed high densities of cells expressing PD-1 (median, 39.53 cells/mm^2^) and PD-L1 (median, 41.935 cells/mm^2^), as well as CD3 + LAG3 + (median, 229.81 cells/mm^2^), CD3 + ICOS + (median, 36.19 cells/mm^2^), CD3 + OX40 + (median, 12.07 cells/mm2), CD3 + B7-H3 + (median, 8.86 cells/mm^2^), and CD3 + TIM3 + cells (median, 6.64 cells/mm^2^). We also observed high quantities of B-cells expressing ICOS (median, 7.44 cells/mm^2^), OX40 (median, 4.72 cells/mm^2^), and LAG3 (median, 31.10 cells/mm^2^). Checkpoints IDO-1 and VISTA in T-cells and TIM3 in B-cells were observed in low densities. Other T and B subcellular populations were identified using co-expression of the markers across panels, as shown in Fig. 2, illustrating the variability of T-cell and B-cell phenotypes in NSCLC, in particular with checkpoint inhibitors. In addition, a total of 48 T-cell and B-cells phenotypes were detected using panels 1, 2, 3, and 4. In ADC specimens (compared with SCC specimens), we observed significantly higher densities of CD3 + CD8 + cytotoxic T-cells, CD3 + CD45RO + memory T-cells, CD3 + CD8 + CD45RO + cytotoxic memory T-cells, CD3 + IDO-1 + T-cells, and CD3 + TIM3 + cells. However, very low densities of CD3 + PD-L1 + cells were observed overall; this cell phenotype was present at higher densities in SCC than in ADC (Supplementary Table 2, Supplementary Fig. 1).

### Macrophages and Myeloid-Derived Suppressor Cell (MDSC) phenotypes

Eleven myeloid cell populations, including tumor-associated macrophages (TAMs), type II TAMs, and MDSCs, were observed in panel 5 (Fig. 2). High densities of CD68 + TAMs (median, 318.32 cells/mm^2^), CD68 + CD11b + myeloid dendritic cells (median, 231.11 cells/mm^2^), CD66b + PMNs (median, 83.79 cells/mm^2^), CD66b + CD11b + immature PMNs (median, 33.58 cells/mm^2^), and CD11b + CD66b + CD33 + granulocytic myeloid-derived suppressor cells (MDSC-PMNs; median, 11.44 cells/mm^2^) were predominantly observed in this NSCLC cohort, suggesting an important myeloid-suppressive component in these tumors. Other myeloid cells such as CD68 + Arg-1+ type II TAMs, CD68 + Arg-1 + CD11b + immature type II TAMs, and CD11b + Arg-1 + CD14 + CD33 + monocytic MDSCs were observed, but in very low densities (Supplementary Table 2). Significantly higher densities of CD68 + CD11b + myeloid dendritic cells, as well as CD66b + PMNs and CD11b + CD66b + immature PMNs, were observed in SCC than in ADC (Supplementary Fig. 1, Supplementary Table 2).

### Patterns of cellular distribution in the tumor microenvironment

**Fig. 3:**
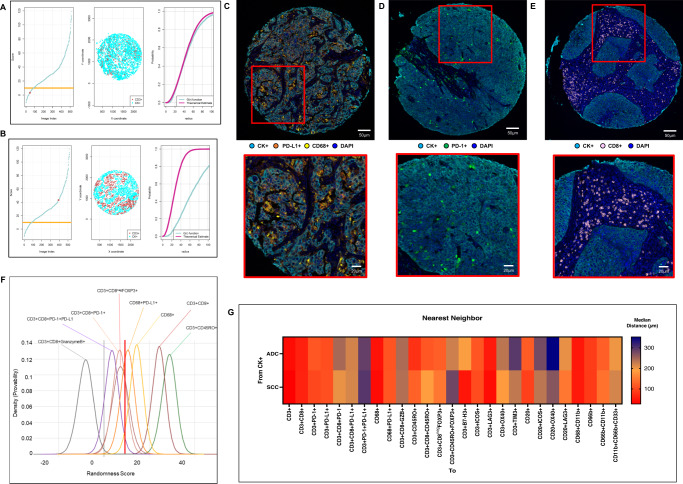
Nearest neighbor distance G function and theoretical Poisson curve score graphs showing different cellular patterns of distance from cytokeratin + cells (malignant cells) to CD3 + T-cells, and heat map representing distance patterns by histologic type. **A**, **B** Representative example of the scoring system across tissue specimens and threshold to be considered part of the mixed (**A**) or unmixed (**B**) pattern. Graphs represent the scoring system (left), point pattern distributions related to the major T-cell population (middle), and G function and theoretical Poisson curve area (right). Composite spectral mixing images from multiplex immunofluorescence (mIF; 20× magnification, scale bars represent 50 µm on each image) showing a representative image of mixed pattern of macrophages PD-L1 expression (**C**) and PD-1+ antigen experience T-cells (**D**), in the *bottom* inside detail of the pattern (mIF; 40× magnification, scale bars represent 20 µm on each image). Unmixed pattern of CD8 + cytotoxic T-cells (**E**) and in their *bottom* inside detail of the pattern (mIF; 40× magnification, scale bars represent 20 µm on each image). **F** Model interaction based on the G function and theoretical Poisson curve score shows two groups of interaction between the most critical cell phenotypes observed and malignant cells. Cell phenotypes with a score of −10 to 10 were characterized as having a mixed/heterogeneous pattern indicating more interaction with malignant cells, and cell phenotypes with a score of >10 were characterized as having an unmixed/clustering pattern indicating less interaction with malignant cells. **G** Median distance heat map representing the 27 most common tumor-associated immune cells near malignant cells (CK + ) across the multiplex immunofluorescence panels in adenocarcinomas (ADCs = 142 samples) and squamous cell carcinomas (SCCs = 83), data from 225 samples was used. Experiments and quantifications related to the presented results were conducted once. Graphs and heat map were generated using R studio software version 3.6.1. mIF images were generated using Vectra-Polaris 1.0.13 scanner system and InForm 2.4.8 image analysis software (Akoya Biosciences). (Source data is provided as a source data file).

After comparing the empirically derived G function curves from T-cells, B-cells, PMNs, and macrophages (as critical markers) with the theoretical Poisson function curve, we identified two patterns of distribution: mixed or heterogeneous (score ranging from −10 to 10; Fig. 3A) and unmixed or clustering (score >10; Fig. 3B), independent of histologic type (ADC or SCC). To avoid bias in this analysis, we used only the most abundant cell phenotypes (i.e., median >2 cells/mm^2^) to identify these patterns. We found that 12 of 26 expected cell phenotypes had a predominant mixed pattern (Fig. 3 C-D, examples), and 13 had a dominant unmixed pattern (Figure E, example) in both ADC and SCC (Table 1). Only CD3 + CD45RO + FOXP3 + memory regulatory T-cells showed significantly different patterns between ADC and SCC (i.e., mixed pattern in ADC and unmixed in SCC; Table 1). In addition, we identified two groups of cell phenotypes using this approach: cells in direct contact with malignant cells (suppressive T-cells and MDSC-PMNs) showing a mixed pattern, and cells with less contact with malignant cells (including macrophages, suppressor macrophages, cytotoxic T-cells, and memory T-cells; Fig. 3F) showing an unmixed pattern. This suggests that in most tumor microenvironments, immunosuppressive cell populations have the most direct contact with malignant cells but are present in lower densities than other types of cells. Interestingly, most cases showed CD20 + B-cells in a mixed pattern, suggesting no activation of these cells into tertiary lymphoid structures (TLS) where the B-cells are distributed in clusters.

**Table 1:** Patterns of cellular distribution according to histologic type (*n* = 225)

Panel	Phenotype	Pattern of malignant cells^a^			*P*†
		NSCLC	Adenocarcinoma	Squamous cell carcinoma	
1	CD3 +	27.96	25.05	38.86	0.942
	CD3 + CD8 +	25.81	24.03	31.92	0.902
	CD3 + PD-1+	16.53	12.17	23.92	0.132
	CD3 + PD-L1 +	20.19	19.45	22.64	0.386
	CD3 + CD8 + PD-1+	3.20	2.89	4.79	0.996
	CD3 + CD8 + PD-L1 +	11.07	10.82	13.02	0.769
	CD68 +	17.50	15.55	21.86	0.334
	CD68 + PD-L1 +	9.33	8.10	9.81	0.889
2	CD3 +	27.30	22.98	37.84	0.933
	CD3 + CD8 +	29.88	27.20	37.36	0.644
	CD3 + CD8 + GZB +	0.36	0.04	0.89	0.372
	CD3 + CD45RO +	35.84	30.14	48.40	0.933
	CD3 + CD8 + CD45RO +	31.10	25.35	41.40	0.294
	CD3 + CD8^neg^FOXP3 +	29.55	26.75	37.41	0.428
	CD3 + CD45RO + FOXP3 +	24.30	8.92	14.80	<0.001
3	CD3 +	29.14	28.38	32.00	0.260
	CD3 + B7-H3 +	26.49	25.31	28.83	0.816
	CD3 + PD-L1 +	30.27	28.75	32.32	0.230
4	CD3 +	21.00	20.33	28.00	0.633
	CD3 + ICOS +	12.00	11.59	14.00	0.647
	CD3 + LAG3 +	22.30	19.03	29.50	0.633
	CD3 + OX40 +	6.35	4.84	9.50	0.232
	CD3 + TIM3 +	4.07	5.22	3.00	0.261
	CD20 +	6.05	4.14	9.00	0.314
	CD20 + ICOS +	4.11	2.15	7.00	0.342
	CD20 + OX40 +	3.00	2.82	3.50	0.954
	CD20 + LAG3 +	10.89	8.95	15.50	0.280
5	CD68 +	19.64	17.31	24.76	0.884
	CD68 + CD11b +	21.84	18.95	26.81	0.365
	CD66b +	3.54	2.13	6.32	0.186
	CD11b + CD66b +	4.00	2.54	8.50	0.720
	CD11b + CD66b + CD33 +	3.00	1.96	6.00	0.113

**Note:** NSCLC, non-small cell lung cancer. For determining the patterns of distribution, 26 relevant cell phenotypes with densities >2 cell/mm^2^ were considered in the analysis according to NSCLC and histologic types, totalizing 78 results. In addition, the data was used to correlate with clinicopathologic variables.

^a^A score of −10 to 10 indicates a mixed pattern, and a score >10 indicates an unmixed pattern.

† *P-*values indicate comparison between adenocarcinoma and squamous cell carcinoma using Kruskal-Wallis test and un-adjusted *P*-values.

### Cellular spatial distances of TAICs from malignant cells in the tumor microenvironment

Using the median nearest neighbor distance from malignant cells to various primary TAICs, we observed that in NSCLC, the median distance from malignant cells to CD3 + T-cells was 36.46 µm; to CD20 + B-cells, 104.61 µm; to CD68 + macrophages, 42.24 µm; and to CD66b + PMNs, 87.24 µm (Table 2). We characterized the distances of subfamilies inside these median radii as close to malignant cells and those outside these median radii as far from malignant cells. The distances of 26 cell phenotypes were measured and analyzed to malignant cells and malignant cells expressing checkpoint markers. Using this dichotomy, we observed that T-cells expressing inhibitory checkpoint markers, such as PD-L1 and B7-H3, which are abundant T-cell phenotypes, were relatively close to malignant cells expressing checkpoint inhibitors PD-L1, B7-H3, B7-H4, IDO-1, and OX40 (Supplementary Table 3). CD3 + PD-L1 + cells were observed next to B7-H4 + malignant cells (median, 26.67 µm) and IDO-1+ malignant cells (median, 24.07 µm). Although the median distance of CD68 + macrophages from malignant cells was 42.24 µm, CD68 + macrophages and PD-L1 + macrophages were closer to PD-L1^neg^ malignant cells (median, 12.90 µm and 51.89 µm, respectively) than to PD-L1 + malignant cells (median, 42.31 µm and 119.71 µm, respectively), suggesting that inhibitory signals are closer to PD-L1^neg^ malignant cells than to PD-L1 + malignant cells.

**Table 2:** Median distances from malignant cells for various cell phenotypes according to histologic type (*n* = 225)

Panel	Phenotype	Median distance from malignant cells, µm			P*
		NSCLC	Adenocarcinoma	Squamous cell carcinoma	
1	CD3 +	29.28	26.88	38.38	0.002
	CD3 + CD8 +	59.66	58.62	61.36	0.003
	CD3 + PD-1+	104.31	113.53	99.03	0.181
	CD3 + PD-L1 +	105.56	105.02	105.56	0.870
	CD3 + CD8 + PD-1	220.89	245.71	203.72	0.163
	CD3 + CD8 + PD-L1 +	218.69	218.69	216.45	0.551
	CD68 +	42.01	44.52	37.57	0.168
	CD68 + PD-L1 +	113.40	111.04	113.41	0.778
2	CD3 +	30.42	27.66	42.28	<0.001
	CD3 + CD8 +	60.40	54.07	78.40	<0.001
	CD3 + CD8 + GZB +	240.62	243.26	238.91	0.576
	CD3 + CD45RO +	91.15	75.96	125.49	<0.001
	CD3 + CD8 + CD45RO +	140.69	114.36	186.28	0.001
	CD3 + CD8^neg^FOXP3 +	135.08	121.99	147.59	0.002
	CD3 + CD45RO + FOXP3 +	252.20	221.84	281.49	0.013
3	CD3 +	28.44	22.55	48.79	<0.001
	CD3 + B7-H3 +	57.52	191.96	48.42	0.154
	CD3 + PD-L1 +	125.09	135.49	115.57	0.203
4	CD3 +	36.46	29.56	43.22	0.023
	CD3 + ICOS +	105.15	113.21	102.80	0.362
	CD3 + LAG3 +	46.46	53.22	39.56	0.023
	CD3 + OX40 +	201.04	233.01	184.20	0.019
	CD3 + TIM3 +	256.75	295.88	230.01	0.007
	CD20 +	104.61	118.82	91.79	0.060
	CD20 + ICOS +	257.72	277.59	232.22	0.084
	CD20 + OX40 +	305.35	353.03	266.19	0.041
	CD20 + TIM3 +	448.24	453.10	444.30	0.436
5	CD68 +	42.24	43.05	40.60	0.513
	CD68 + CD11b +	51.81	51.13	52.30	0.613
	CD66b +	87.24	87.98	85.22	0.385
	CD11b + CD66b +	133.49	121.79	141.08	0.358
	CD11b + CD66b + CD33 +	214.99	199.89	217.28	0.864

**Note:** NSCLC, non-small cell lung cancer. For determining the median distances, 26 relevant cell phenotypes with densities >2 cell/mm^2^ were considered in the analysis according to NSCLC and histologic types, totalizing 78 results. In addition, the data was used to correlate with clinicopathologic variables.

*P-values indicate comparison between adenocarcinoma and squamous cell carcinoma using Kruskal-Wallis test and un-adjusted *P*-values.

Additionally, upon examining the radii from malignant cells to other distinct TAIC phenotypes, we observed that CD3 + CD8 + cytotoxic T-cell, CD3 + CD45RO + memory T-cell, CD3 + CD8^neg^FOXP3 + regulatory T-cell, B-cell, and myeloid cell subpopulations were located relatively far from the overall malignant cells compared with the other cell populations described below, as shown in the heat map in Fig. 3G. CD3 + CD8^neg^FOXP3 + regulatory T-cells were close to CD3 + CD8 + cytotoxic T-cells (median, 26.73 µm), CD3 + CD45RO + memory T-cells (median, 40.83 µm), and CD3 + CD8 + CD45RO + cytotoxic memory T-cells (median, 43.05 µm), suggesting a possible inhibitory action from this T-cell phenotype to cytotoxic and memory T-cells (Supplementary Table 2). Furthermore, we observed that cytotoxic T-cells, memory T-cells, cytotoxic memory T-cells, regulatory T-cells, and effector memory T-cells were significantly closer to malignant cells in ADC than in SCC. In contrast, a considerably closer distance from malignant cells to T-cells expressing LAG3, OX40, and TIM3 and B-cells expressing OX40 and LAG3 was observed in SCC compared with ADC.

### Cellular immunologic distribution landscape

By combining the cellular distribution patterns with the median distances of TAICs from malignant cells, we identified four cellular immunologic distribution groups for each cell phenotype study across the panels:

Group 1. Mixed pattern with close median distances to the malignant cells

Group 2. Mixed pattern with long median distances to the malignant cells

Group 3. Unmixed pattern with close median distances to the malignant cells

Group 4. Unmixed pattern with long median distances to the malignant cells

Given that CD3 + T-cells were the predominant cell population in the tumor microenvironment and across the panels, we observed that group 4 (unmixed pattern with long median distances to the malignant cells) was the predominant group, which was observed in 36.8% of the NSCLC specimens; followed by group 2 (mixed pattern with long median distances to malignant cells), which was observed in 30.6% of the specimens; group 1 (mixed pattern with close median distances to the malignant cells), observed in 23.6% of the specimens; and finally group 3 (unmixed pattern with close median distances to the malignant cells), observed in 9.0% of the specimens. Furthermore, we characterized the cellular phenotype densities related to these groups (Table 3). We found that group 2 contained the highest cellular densities of CD3 + T-cells, CD3 + CD8 + cytotoxic T-cells, CD3 + CD8 + GZB + activated cytotoxic T-cells, and CD3 + CD45RO + memory T-cells. However, we also observed higher densities of CD3 + CD8^neg^FOXP3 + regulatory T-cells, CD3 + CD45RO + FOXP3 + regulatory/memory T-cells, CD3 + PD-1+ antigen-experienced T-cells, and CD3 + CD8 + PD-1+ antigen-experienced cytotoxic T-cells in group 2 than in other groups, suggesting that group 2 represents inflamed tumors containing a diversity of cell phenotypes, both immunoprotective and immunosuppressive.

**Table 3:**
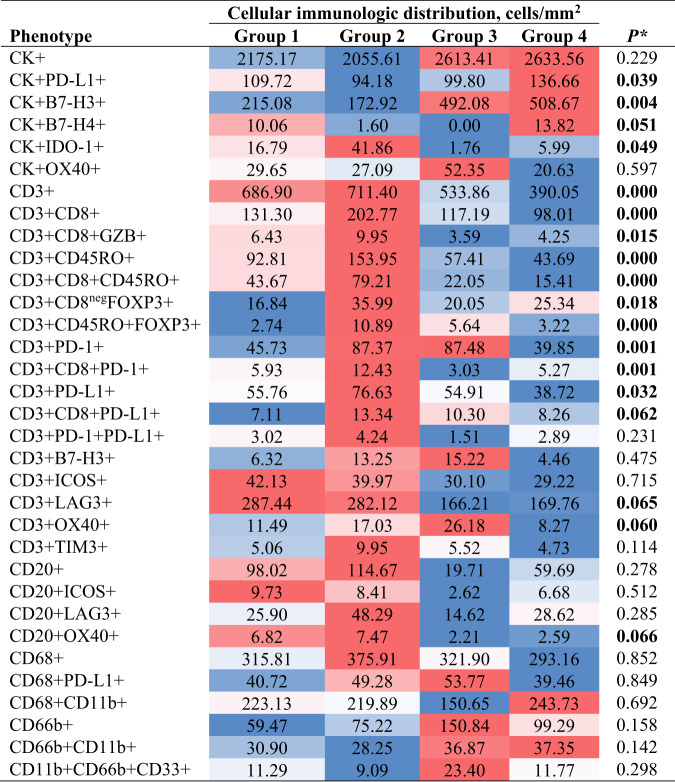
Cell phenotype densities according the cellular immunologic distribution based on CD3 + T-cells in non-small cell lung cancer (*n* = 225)

**Note**: * Boldface indicates statistically significant difference using Kruskal–Wallis 1-way ANOVA test with un-adjusted *P*-values between groups. A total of 33 cell phenotypes are showing across the four different cellular immunologic distribution groups. Variations of color from red (inflamed) to blue (Cold) indicate variations of median densities of different cell phenotypes from high to low densities among the groups.

Furthermore, the predominant immune checkpoint expressed by malignant cells in group 2 was IDO-1. In contrast, group 4, the most predominant group in our cohort, showed significantly lower densities of the cell phenotypes described in group 2. The highest densities of malignant cells expressing B7-H3, B7-H4, and PD-L1 were observed in group 4, suggesting that group 4 represents “cold” tumors driven by the inhibitory checkpoint markers expressed by malignant cells. Group 1 and group 3 had cellular densities falling in between those observed in groups 2 and 4; group 3 was the least commonly observed pattern in our cohort. Group 1 had higher expression of PD-L1 by malignant cells than did group 3, and group 3 had higher expression of B7-H3 by malignant cells than did group 1.

### Association of cellular distribution patterns and spatial metrics of TAICs with clinical variables

To study associations between clinical variables and cellular patterns of distribution and spatial cellular distances from malignant cells, we used the comparison of the G function curve with the theoretical Poisson curve (26 patterns of cell phenotypes) and the median distance from malignant cells to various cell phenotypes (128 distances).

**Fig. 4:**
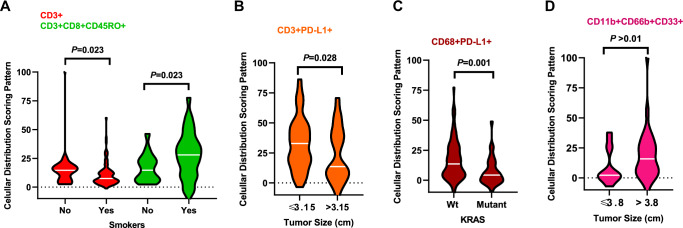
Violin plots showing associations between patterns of immune cell distribution and clinicopathologic features. Significant cellular distribution scoring patterns between immune cells and malignant cells are shown by (**A**) smoker status, (**B**) tumor size, and (**C**) *KRAS* mutation status for lung adenocarcinoma (*n* = 142) specimens. **D** Significant cellular distribution scoring patterns between malignant cells and immune cells are shown by tumor size for lung squamous cell carcinoma (*n* = 83) specimens. Violin plots showing the median bar value, lower adjacent value and outside points. Kruskal–Wallis test was used in **A**–**D** comparisons between groups. Data from 225 samples was used. Graphs were generated using GraphPad Prism v.9.0.0 using the 26 cell phenotypes distribution patterns and the relevant clinicopathologic information using un-adjusted *P*-values. (Source data is provided as a source data file).

Cellular patterns of distribution in ADC specimens from patients who were smokers revealed a significant mixed pattern of CD3 + T-cells compared with specimens from patients who were nonsmokers. In contrast, a significant unmixed pattern of CD3 + CD8 + CD45RO + cytotoxic memory T-cells was observed in specimens from smokers compared with nonsmokers (Fig. 4A). Compared with smaller tumors (≤3.15 cm; the median within the group was used as a cutoff for tumor size comparisons), ADC tumors >3.15 cm showed a significant mixed pattern of CD3 + PD-L1 + cells (Fig. 4B). ADC *KRAS*-mutant tumors showed a significant mixed pattern of CD68 + PD-L1 + cells compared with wild-type tumors (Fig. 4C). In SCC, tumors >3.8 cm showed a significant unmixed pattern of CD11b + CD66b + CD33 + MDSC-PMNs compared with tumors ≤3.8 cm (Fig. 4D).

**Fig. 5:**
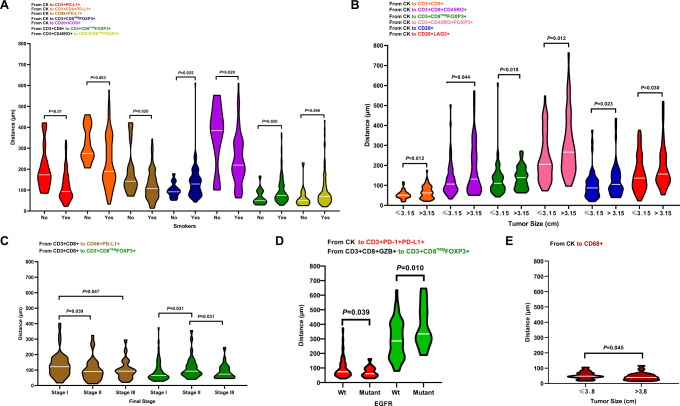
Violin plots showing the significant associations between distances of immune cell populations from malignant cells and clinicopathologic features. Significant differences in distance between malignant cells and immune cells are shown by (**A**) smoker status, (**B**) tumor size, (**C**) final stage, and (**D**) mutation status in lung adenocarcinoma (*n* = 142) specimens. **E** Significant differences in distances between malignant cells and immune cells by tumor size are shown for lung squamous cell carcinoma (*n* = 83) specimens. Violin plots showing the median bar value, lower adjacent value and outside points. Kruskal-Wallis test was used in **A** to **E** comparisons between groups. Data from 225 samples was used. Graphs were generated using GraphPad Prism v.9.0.0 using the 128 measurements from malignant cells to different tumor-associated immune cells (TAICs) and between important TAICs and the relevant clinicopathologic information using un-adjusted *P*-values. (Source data is provided as a source data file).

In ADC specimens from smokers, using the distance analysis, we observed a significant close median distance from malignant cells to suppressive TAIC phenotypes such as PD-L1 + T-cells, CD3 + CD8 + cytotoxic T-cells expressing PD-L1, macrophages expressing PD-L1, and ICOS + B-cells compared with nonsmokers. In ADC specimens from nonsmokers, CD3 + CD8^neg^FOXP3 + regulatory T-cells were located significantly close to malignant cells, close to CD3 + CD8 + cytotoxic T-cells, and close to CD3 + CD45RO + memory T-cells than non-smokers (Fig. 5A).

Among ADC specimens, tumors ≤3.15 cm showed CD3 + CD8 + cytotoxic T-cells, CD3 + CD8 + CD45RO + FOXP3 + cytotoxic memory T-cells, CD3 + CD8^neg^FOXP3 + regulatory T-cells, CD3 + CD45RO + memory regulatory T-cells, B-cells, and LAG3 + B-cells located significantly closer to malignant cells than in the larger tumors (Fig. 5B). Significantly close distances from PD-L1 + macrophages to cytotoxic CD3 + CD8 + T-cells were observed in stage II and III specimens compared with stage I specimens. Moreover, significantly close distances from CD3 + CD8^neg^FOXP3 + regulatory T-cells to cytotoxic CD3 + CD8 + T-cells were observed in stage I and III compared with stage II ADC specimens (Fig. 5C).

*EGFR*-mutant tumors had significantly closer distances from CD3 + PD-1+PD-L1 + T-cells to malignant cells and from CD3 + CD8^neg^FOXP3 + regulatory T-cells to CD3 + CD8 + GZB +  activated cytotoxic T-cells than did wild-type tumors in ADC (Fig. 5D).

In SCC, tumors larger than the median (>3.8 cm) showed significantly closer distances from malignant cells to macrophages than did smaller tumors (Fig. 5E).

### Associations between cellular patterns or distances and patient outcomes

**Fig. 6:**
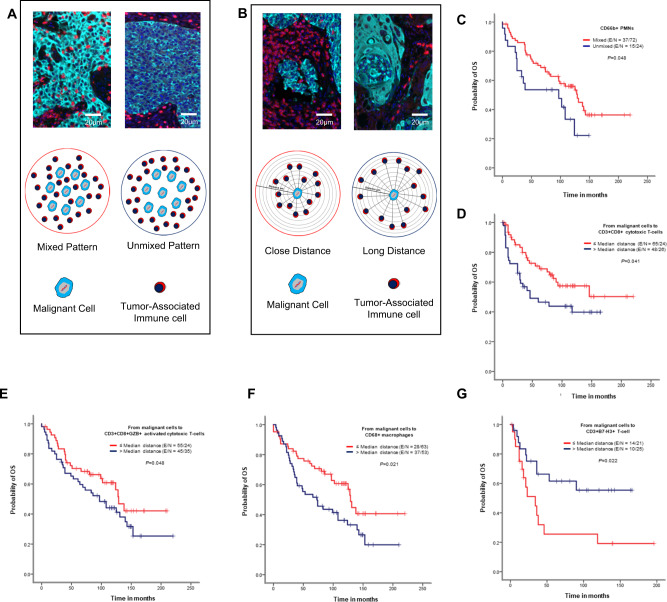
Kaplan–Meier analysis of overall survival (OS) by cellular patterns of distribution and distance from malignant cells to various immune cell subpopulations. **A** Composite spectral mixing images from a detail of multiplex immunofluorescence (mIF; 40× magnification scale bars represent 50 µm on each image) and illustration of the two different patterns of distribution, mixed and unmixed. **B** Composite spectral mixing images from a detail of multiplex immunofluorescence (mIF; 40× magnification, scale bars represent 50 µm on each image) and illustration of the distance metrics from malignant cells to different immune cells. (**C**–**G**) Kaplan-Meier OS curves. Red lines indicate mixed pattern or close (≤median) distances between malignant cells and various cell phenotypes, and blue lines indicate unmixed pattern or long (>median) distances between malignant cells and various cell phenotypes. **C** Patients with CD66b + granulocytes (PMNs) with a mixed pattern had better OS than those with an unmixed pattern in lung adenocarcinoma specimens. Close (≤median) distances from malignant cells to (**D**) CD3 + CD8 + cytotoxic T-cells, (**E**) CD3 + CD8 + GZB + activated cytotoxic T-cells, and (**F**) CD68 + macrophages and long (>median) distances from malignant cells to B7-H3 + T-cells were associated with better OS in lung adenocarcinoma specimens using un-adjusted *P*-values. Data from 225 samples was used. Experiments and quantifications related to the presented results were conducted once. The images were generated using the Vectra-Polaris 1.0.13 scanner system and InForm 2.4.8 image analysis software (Akoya Biosciences), and Kaplan–Meier curves and log-rank test were used and generated by the R studio software version 3.6.0. using the distribution patterns and distances from the 26-cell phenotypes with un-adjusted *P*-values. (Source data is provided as a source data file).

We next examined whether cellular distribution patterns of 26 TAICs (Fig. 6A) or TAIC distances from malignant cells (Fig. 6B) were associated with patient outcomes. Univariate analysis of cellular distribution patterns showed that the unmixed pattern of CD66b + PMNs was associated with worse OS than mixed patterns in ADC (Fig. 6C). In SCC, the mixed pattern in CD3 + PD-L1 + T-cells and CD3 + CD8 + GZB + activated cytotoxic T-cells was associated with better RFS than the unmixed pattern (Supplementary Fig. 2A, B). The multivariable Cox proportional hazards model adjusted for histologic type, smoking status, tumor size, *KRAS* mutation status, and *EGFR* mutation status, as shown in Supplementary Table 4, showed that patients with large tumors (>3.8 cm), a *KRAS* mutation, and a mixed CD3 + TIM3 + pattern had worse OS than patients with tumors ≤1.5 cm, wild-type *KRAS*, and an unmixed CD3 + TIM3 + pattern.

Using distances from malignant cells to TAICs (Fig. 6B), univariate analysis in our cohort showed that long distances of CD66b + PMNs from malignant cells in ADC was associated with better RFS than close distances (Supplementary Fig. 2C). In SCC, long distances from CD3 + PD-L1 + T-cells to malignant cells and close distances from CD3 + ICOS + T-cells to malignant cells were associated with better RFS (Supplementary Fig. 2D, E). Furthermore, close distances from CD3 + CD8 + cytotoxic T-cells, CD3 + CD8 + GZB + activated cytotoxic T-cells, and CD68 + macrophages to malignant cells were associated with better OS than long distances in ADC (Fig. 6D–F). In contrast, close distances from CD3 + B7-H3 + T-cells to malignant cells were associated with worse OS than long distances (Fig. 6G).

As shown in Supplementary Table 5, the Cox regression model showed that ADC (compared with SCC), tumor size <1.5 cm, wild-type *KRAS*, and close distances from malignant cells to overall CD3 + T-cells, CD3 + CD8 + GZB + activated cytotoxic T-cells, CD3 + CD8 + CD45RO + memory cytotoxic T-cells, and overall CD20 + B-cells were associated with better OS. In addition, close distances from malignant cells to CD3 + PD-1 + , CD3 + PD-L1 + , CD3 + TIM3 + , CD3 + ICOS + , and CD66b + CD11b + cells, observed in low densities in our cohort, were also associated with better OS in the multivariable analysis. Factors associated with worse OS included nonsmoker status, wild-type *EGFR* mutation, and close distance from malignant cells to CD3 + CD8^neg^FOXP3 + regulatory T-cells, CD3 + CD45RO + FOXP3 + memory/regulatory T-cells, CD3 + CD8 + cytotoxic T-cells expressing PD-L1, CD3 + T-cells expressing OX40 + , LAG3 + cells, and CD68 + macrophages expressing PD-L1. Furthermore, we included cellular densities from selected T-cell phenotypes, the patterns of distribution, and distances relative to malignant cells in a multivariate analysis, as shown in Table 4. This analysis revealed the lowest densities of overall T-cell CD3 + , cytotoxic T-cells CD3 + CD8 + , cytotoxic antigen-experienced T-cells CD3 + CD8 + PD-1 + , and T-cell expression PD-L1; and mixed pattern of memory T-cells CD3 + CD45RO + and T-cells antigen-experienced PD-L1 + , and closed distances to malignant cells of regulatory T-cells CD3 + CD8^neg^FOXP3 + , memory/regulatory T-cells CD3 + CD45RO + FOXP3 + , and T-cells expressing PD-L1 as factors associated to worse OS. In contrast, patients with smaller tumors ≤1.5 cm, lowest densities of memory/regulatory T-cells CD3 + CD45RO + FOXP3 + and antigen T-cells antigen-experienced PD-L1 + , and mixed pater of overall T-cells CD3 + , and close distances of activated cytotoxic T-cells CD3 + CD8 + GZB + relatively to the malignant cells as factors associated with better survival. We also examined the effects of the four groups of cellular immunologic distribution, combining cellular patterns of TAIC distribution with TAIC distances from malignant cells, on patient outcomes, adjusted for clinicopathologic characteristics (Supplementary Table 6). The Cox proportional hazards regression model adjusted for histologic type, smoking status, tumor size, *KRAS* mutation status, and *EGFR* mutation status showed that factors associated with better OS included nonsmoker status, wild-type *EGFR*, and CD3 + T-cells and CD3 + CD8 + CD45RO + cytotoxic/memory T-cells in an unmixed pattern with close median distances to malignant cells (group 3). In contrast, CD3 + CD8^neg^FOXP3 + unmixed pattern with close median distances to malignant cells (group 3) and mixed pattern with long median distances to malignant cells (group 2) and CD3 + PD-1+PD-L1 + unmixed pattern with close median distances to malignant cells (group 3) were associated with worse OS, suggesting that the pattern of distribution and distance to malignant cells can influence survival.

**Table 4:** Cox proportional hazards regression model of overall survival in patients with non-small cell lung cancer compared high and low densities, close with long distances and mixed with unmixed pattern relative to malignant cells, adjusted for clinicopathologic variables

Variable	B	SE	Wald	HR	95% CI for Exp(B)	P*
Histologic type (ADC vs SCC)	−1.103	1.081	1.042	0.332	0.040–2.759	0.307
Smoker (no vs yes)	1.702	1.938	.772	5.485	0.123–244.607	0.380
Tumor size (≤1.5 cm vs >3.8 cm)	−2.328	0.854	7.432	0.098	0.018–0.520	**0.006**
*KRAS* (wild-type vs mutant)	1.452	0.981	2.191	4.273	0.625–29.231	0.139
*EGFR* (wild-type vs mutant)	−1.411	1.207	1.367	.244	0.023–2.597	0.242
Low vs high densities						
CD3 +	3.377	0.996	11.506	29.289	4.161–206.159	**0.001**
CD3 + CD8 +	2.882	1.126	6.545	17.847	1.962–162.338	**0.011**
CD3 + CD8 + GZB +	0.524	1.296	0.164	1.689	0.133–21.429	0.686
CD3 + CD45RO +	−2.725	1.669	2.665	0.066	0.002–1.727	0.103
CD3 + CD8 + CD45RO +	0.644	1.288	0.250	1.905	0.153–23.766	0.617
CD3 + CD8^neg^FOXP3 +	0.913	0.670	1.854	2.491	0.670–9.268	0.173
CD3 + CD45RO + FOXP3 +	−4.203	1.485	8.008	0.015	0.001–0.275	**0.005**
CD3 + PD-1+	−0.142	0.798	0.032	0.868	0.182–4.143	0.859
CD3 + CD8 + PD-1+	4.311	1.405	9.414	74.550	4.746–1170.936	**0.002**
CD3 + PD-L1 +	8.036	1.729	21.612	3090.038	104.373–91482.972	**0.000**
CD3 + CD8 + PD-L1 +	0.486	1.224	0.157	1.625	0.148–17.880	0.692
CD3 + PD-1+PD-L1 +	−3.646	1.074	11.519	0.026	0.003–0.214	**0.001**
Mixed vs unmixed pattern						
CD3 +	−3.073	1.124	7.471	0.046	0.005–0.419	**0.006**
CD3 + CD8 +	0.995	1.241	.643	2.704	0.238–30.771	0.423
CD3 + CD8 + GZB +	−0.593	.800	.551	.552	0.115–2.648	0.458
CD3 + CD45RO +	3.454	1.152	8.990	31.623	3.307–302.378	**0.003**
CD3 + CD8 + CD45RO +	0.526	1.196	0.193	1.692	0.162–17.628	0.660
CD3 + CD8^neg^FOXP3 +	7.917	61.229	0.017	2743.024	0.000–3.601E + 55	0.897
CD3 + CD45RO + FOXP3 +	−1.087	0.844	1.661	0.337	0.065–1.761	0.197
CD3 + PD-1+	−1.861	1.136	2.683	0.155	0.017–1.442	0.101
CD3 + CD8 + PD-1+	1.004	0.775	1.679	2.729	0.598–12.464	0.195
CD3 + PD-L1 +	−.508	1.065	0.228	0.602	0.075–4.855	0.633
CD3 + CD8 + PD-L1 +	1.288	1.139	1.279	3.624	0.389–33.763	0.258
CD3 + PD-1+PD-L1 +	3.291	1.346	5.976	26.874	1.920–376.094	**0.014**
Close vs long distance from malignant cells						
CD3 +	−0.174	1.009	0.030	0.840	0.116–6.071	0.863
CD3 + CD8 +	−1.376	1.253	1.206	0.253	0.022–2.945	0.272
CD3 + CD8 + GZB +	−2.967	1.383	4.604	.051	0.003–0.773	**0.032**
CD3 + CD45RO +	−1.248	1.281	0.949	0.287	0.023–3.537	0.330
CD3 + CD8 + CD45RO +	1.043	1.273	0.672	2.839	0.234–34.388	0.412
CD3 + CD8^neg^FOXP3 +	2.446	1.018	5.773	11.538	1.569–84.822	**0.016**
CD3 + CD45RO + FOXP3 +	3.032	1.150	6.956	20.735	2.179–197.331	**0.008**
CD3 + PD-1+	1.461	1.100	1.763	4.311	0.499–37.260	0.184
CD3 + CD8 + PD-1+	1.754	1.012	3.007	5.778	0.796–41.956	0.083
CD3 + PD-L1 +	5.445	1.706	10.188	231.550	8.178–6556.148	**0.001**
CD3 + CD8 + PD-L1 +	2.316	1.920	1.455	10.134	0.235–436.512	0.228
CD3 + PD-1+PD-L1 +	−2.021	1.354	2.229	0.132	0.009–1.882	0.135

**Note:** B unstandardized regression weight, SE multiple linear regression, *Wald* Wald test, *HR* hazard ratio, *CI* confidence interval, *ADC* adenocarcinoma, *SCC* squamous cell carcinoma, *GZB* granzyme B.

*Boldface indicates statistically significant difference using Cox proportional-hazards model with un-adjusted *P*-values for clinicopathologic variables.

The table shows the analysis of densities, and pattern of cellular distribution and distances relative to malignant cells to the 12 relevant cell phenotypes of the multiplex immunofluorescence panels adjusted by clinicopathologic features.

## Discussion

In the current study, we analyzed a TMA set containing tumor specimens obtained from a large cohort of patients with stage I-III NSCLC using 23 markers, including T-cell, B-cell, immune checkpoint, and myeloid cell markers, placed in 5 mIF panels. Cord plots and UMAP plots based on marker co-expression showed the diversity of cell phenotypes across different panels in NSCLC. We found that malignant cells expressing B7-H3 were most commonly observed, followed by malignant cells expressing PD-L1, OX40, B7-H4, and IDO-1. These checkpoints have adverse regulatory functions over T lymphocytes^25–28^. Other immune checkpoints observed in our cohort, such as OX40 and ICOS, have co-stimulatory signals for T-cell activation in normal and pathologic conditions^29–31^. Cord plots and UMAP plots showed that various TAICs and malignant cells could express these checkpoints reinforce our preliminary results using individual markers in a similar cohort^32,33^. Although it was thought for a long time that OX40 expression was restricted to activated conventional T-cells, other TAICs, including malignant cells, have since been shown to express this marker^34^, as was also shown in our preliminary report^16^.

Overall, we observed higher densities of immune checkpoint markers in SCC than in ADC, particularly PD-L1, B7-H3, and B7-H4, showing that these immune checkpoints are predominantly expressed in solid tumors^35^, creating a more immunosuppressive microenvironment. It is clear that in NSCLC, these immune checkpoint pathways are expressed simultaneously and are an essential mechanism of immune resistance against T-cell response^36,37^. Our findings in the current study confirm our previous findings^16,33^ showing that malignant cells could express more than one checkpoint marker simultaneously, indicating that lung tumors can use more than one pathway to avoid the immune system^38,39^. Immune checkpoints are essential regulators of the immune system, initiating a productive immune response, preventing the onset of autoimmunity, or using tumors to avoid the immune system^40^. In agreement with other studies^31^, our results show that TAICs and malignant cells essentially drive these suppressive pathways. This knowledge of simultaneous co-expression can guide the study of rational combinations of agents for potential new therapeutic approaches.

Although we observed predominantly CD3 + CD8 + cytotoxic T-cells, CD3 + CD45RO + memory T-cells, CD3 + CD8 + CD45RO + cytotoxic memory T-cells, and CD20 + B-cells in the NSCLC specimens, we also confirmed that TAICs express co-inhibitory and co-stimulatory signatures, including PD-1, LAG3, TIM3, FOXP3, ICOS, and OX40, in higher amounts in SCC than in ADC, as previously reported by our group^32,33^. Similarly, we observed that CD68 + macrophages, CD68 + CD11b + myeloid dendritic cells, and CD66b + PMNs are more predominant in SCC than in ADC. These observations suggest that various immunosuppressive and immunoprotective cells are present in these tumors, possibly reflecting the relation of these cells to other factors such as smoking status, tumor size, mutational status, or chronic obstructive pulmonary disease and their impact on patient survival, which were previously shown by our group and others^32,33,41^. Although myeloid cell phenotypes such as CD68 + Arg-1+ type II macrophages, CD68 + Arg-1 + CD11b + type II immature TAMs, CD66b + CD11b + immature PMNs, Arg-1 + CD33 + CD14 + CD11b + monocytic MDSCs, and CD33 + CD66b + CD11b + MDSC-PMNs were detected in low densities, these were also present, playing their immunosuppressive roles^42^.

Although we identified several immune signatures across the panels in the current study and described densities association with clinicopathologic information using the same cohort, in previous studies^32,33^, we focused this time on the spatial relationships between tumor and immune cells and between immune cells, which may provide insight into prognostic indicators as previously described^16^. In the current study, we thus identified mixed and unmixed cellular distribution patterns, likely related to the dysfunctional signature observed in melanoma tumor tissues^17^. We also observed two groups of cells in relation to malignant cells: an immunosuppressive group, which has a predominant mixed pattern indicating close interaction with malignant cells, and an immunoprotective group, which had an unmixed pattern with apparently less interaction with malignant cells. The immunoprotective group included CD3 + CD8 + cytotoxic T-cells, CD3 + CD8 + CD45RO + cytotoxic memory T-cells, and CD3 + CD45RO + memory T-cells. We observed in our study that overall CD68 + macrophages have a predominant unmixed pattern. On the other hand, CD20 + B-cells showed, in most cases, a mixed pattern, infiltrating throughout the tumor but not forming organized networks or structured as TLS, suggesting a deficiency in orchestrating the activation, maturation, and intratumoral distribution of other immune cells^18^.

Analysis of nearest neighbor median distance from malignant cells showed that CD3 + T-cells and CD68 + macrophages were closer to malignant cells than were CD20 + B-cells and CD66b + PMNs. Although CD3 + CD8 + cytotoxic T-cells were among the most abundant cells, showing higher densities compared with the other cell phenotypes, in both ADC and SCC specimens, not many of these cells were close to malignant cells. In contrast, T-cells expressing PD-L1, B7-H3, B7-H4, IDO-1, and OX40, which were less abundant, were located close to the malignant cells, suggesting that the distance from malignant cells and pattern of distribution, rather than the density of these cells, play a critical role in cancer. Although CD3 + CD45RO + memory T-cells, CD3 + CD8^neg^FOXP3 + regulatory T-cells, B-cells, and most myeloid cells were located relatively far from malignant cells compared with the other T-cell inhibitors, CD3 + CD8^neg^FOXP3 + regulatory T-cells play a solid immunosuppressive role in the tumor environment by releasing inhibitory cytokines^19^, facilitating the action of other cell inhibitors. Cellular distribution patterns and cellular distances are not frequently studied and are not very well understood, but these patterns can give us essential information about tumor tissue biological processes related to different tumor characteristics^20^.

The association of cellular distribution patterns with clinicopathologic features can help us better understand the biological behavior of tumors. For example, we observed that ADC specimens from smokers overall had a mixed pattern of distribution of overall CD3 + T cells and an unmixed pattern of CD3 + CD8 + CD45RO + effector memory T-cells. The largest ADC tumors showed an unmixed pattern of CD3 + PD-L1 + cells. In ADC *KRAS*-mutant tumors, we observed a mixed pattern of CD68 + PD-L1 + cells. In SCC, we observed changes in cellular distribution patterns predominantly in MDSC populations, which showed a mixed pattern of CD11b + CD66b + CD33 + cells in smaller tumors. Spatial metrics showed that ADC specimens from smokers have close median distances from malignant cells to PD-L1 + T-cells and CD3 + CD8 + cytotoxic T-cells expressing PD-L1. This can be interpreted as tobacco’s immunosuppressive effect on the tumor microenvironment^35^. In contrast, ADC specimens from nonsmokers showed that CD3 + CD8^neg^FOXP3 + regulatory T-cells were relatively close to malignant cells and, most importantly, CD3 + CD8 + cytotoxic T-cells and CD3 + CD45RO + memory T-cells were also close to malignant cells, suggesting a different spatial arrangement and maybe inhibitory mechanism than that observed in tumors from smokers. Small tumors in ADC were enriched in inhibitory signals; in particular, CD3 + CD8^neg^FOXP3 + regulatory T-cells, CD3 + CD45RO + FOXP3 + memory regulatory T-cells, IDO-1 + T-cells, IDO-1 + B-cells, and LAG3 + B-cells were relatively close to the malignant cells compared with the distances observed in larger tumors. In SCC, large tumors showed closer distances from malignant cells to CD68 + macrophages and IDO-1 + T-cells than did small tumors. In addition, stage I and III ADCs showed closer proximity of CD3 + CD8^neg^FOXP3 + regulatory T-cells to cytotoxic CD3 + CD8 + T-cells than did stage II tumors, suggesting changes according to the stage of the tumor. Finally, *EGFR*-mutant tumors showed close distances from suppressor cells, such as CD3 + PD-1+PD-L1 + T-cells, to malignant cells and from CD3 + CD8^neg^FOXP3 + regulatory T-cells to CD3 + CD8 + GZB + activated cytotoxic T-cells, explaining in part the non-responsiveness of these types of tumors to immune checkpoint blockade. It is known that most, if not all, malignancies trigger an innate inflammatory response that builds up a pro-tumorigenic microenvironment that can resist treatment^21^. This suggests that the close proximity of immunosuppressive cells to malignant cells may increase the interactions between these cells in NSCLC, as we observed in the current study, which may enable tumors to avoid the immune system.

We found that not only densities but distribution patterns and distances from malignant cells to different cell phenotypes could be associated with outcomes. Limited penetration among malignant cells, indicated by an unmixed pattern of distribution of MDSC-PMNs, was associated with poor RFS, and an unmixed pattern of CD66b + PMNs and MDSC-PMNs was associated with poor OS in ADC. These findings suggest that these cell phenotypes are acting as a barrier, limiting the actions of other activated T-cells. MDSCs are known to suppress T-cell activation and toxicity using various mechanisms^43^. A mixed pattern of CD3 + CD8 + GZB + activated cytotoxic T-cells was associated with better RFS, suggesting that the close proximity of these cells to malignant cells could prevent tumor recurrence in SCC. Furthermore, the Cox proportional hazards model confirmed that patients with a mixed CD3 + TIM3 + T-cell pattern, large tumors, and *KRAS* mutations had worse OS.

Kaplan–Meier curves showed that in ADC, close distances from malignant cells to PMNs and MDSC-PNMs were associated with worse RFS compared with long distances. In SCC, close distances from malignant cells to PD-L1 + T-cells and long distances to ICOS + T-cells were associated with worse RFS. Our data also showed that close distances from CD3 + CD8 + cytotoxic T-cells, CD3 + CD8 + GZB + activated cytotoxic T-cells, and macrophages to malignant cells was associated with better OS than long distances in ADC, suggesting that the cell-to-cell proximity of these cells mitigates the suppressive effect of inhibitory cells, and supporting the findings of Barua et al^22^. We also found that close distance from malignant cells to B7-H3 + T-cells was associated with worse OS. Additionally, the Cox regression model confirmed that close distances from CD3 + CD8^neg^FOXP3 + regulatory T-cells, CD3 + CD45RO + FOXP3 + regulatory/memory T-cells, CD3 + CD8 + cytotoxic T-cells expressing PD-L1, CD3 + OX40 + T-cells, CD3 + LAG3 + T-cells, and CD68 + macrophages PD-L1 + to malignant cells predicted poor prognosis. This suggests that the cellular spatial distribution of specific cell phenotypes is an independent factor associated with poor or better prognosis and can be used to select combinations of therapeutic strategies and determine patient prognosis^20^, not only in NSCLC but also in other cancers^12^. Including T-cell densities, the pattern of distribution, and distances relative to malignant cells in a Cox regression model we identified that densities of cytotoxic T-cells CD3 + CD8 + and distances of activated cytotoxic T-cells CD3 + CD8 + GZB + to malignant cells are important prognostic factors in NSCLC, as previous reports in the literature^23,24^. Finally, by combining patterns of cellular distribution with cellular distances, we identified four groups of cellular immunologic patterns. The predominant group observed in our cohort was group 2, characterized by high densities of T-cell phenotypes but low densities of immune checkpoints expressed by malignant cells, compared with the other groups, suggesting that the group 2 pattern could indicate an inflamed tumor. In contrast, group 4 showed overall the lowest densities of T-cells but highest densities of immune checkpoints expressed by malignant cells, suggesting that the group 4 pattern indicates “cold” tumors. Multivariable analysis showed that a mixed pattern with long distances or an unmixed pattern with close distances from malignant cells to CD3 + CD8^neg^FOXP3 + regulatory T-cells is associated with worse OS, as was an unmixed pattern with close distances from malignant cells to CD3 + PD-1+PD-L1 + cells. Overall, these findings suggest that the location and distance of suppressive cell signatures, such as regulatory T-cells, from malignant cells as identified by Barua et al^22^ and others, i.e., by checkpoint T-cell inhibitors, can contribute to an immunosuppressive microenvironment, which may be responsible for poor prognosis. These findings highlight the importance of better understanding the complex relationships between malignant cells and immune cells in terms of their spatial distribution to direct the study of new therapeutic approaches.

The current study has some limitations. First, although our NSCLC specimens were collected retrospectively, which allowed us a large enough sample to examine varying cell phenotypes, those phenotypes were displaced in different independent mIF panels, which limited the integration of the different cell phenotypes. Second, most of the patients from our cohort were smokers, which can influence the analysis between nonsmokers and smokers. Lastly, our specimens were placed in TMA format, which may induce under- or overrepresentation of the marker levels and spatial distribution owing to tumor heterogeneity.

In summary, our data showed that tumor cells and TAICs could produce multiple inhibitory factors in NSCLC. In studying the spatial distribution of various cellular populations, we could identify other associations between these cells and clinicopathologic variables in surgically resected ADC and SCC specimens. In addition, we identified several associations between specific cellular patterns of distribution and their distances that can negatively or positively influence patient outcomes; however, validation of our findings using a similar cohort of patients is needed.

## Methods

### Tissue specimens and microarray

We examined specimens from 225 patients with stage I-III primary NSCLC, 142 of which were adenocarcinomas (ADCs) and 83 squamous cell carcinomas (SCCs). The patients had not received neoadjuvant therapy and were evaluated and underwent surgical resection at The University of Texas MD Anderson Cancer Center between 1997 and 2012. Available tissue specimens were obtained from the Lung Cancer Specialized Program of Research Excellence tissue bank at University of Texas MD Anderson Cancer Center, following informed written consent obtained from all study participants under protocols approved by the MD Anderson Institutional Review Board (P50CA70907). Tumors were classified using the 8th American Joint Committee on Cancer guidelines^44^. Tissue microarray (TMA) sections were prepared using triplicate 1-mm-diameter cores from formalin-fixed and paraffin-embedded representative tumor blocks^45^. Clinical and pathologic information, including demographic data, age, sex, tobacco history, smoking status, tumor size, tumor stage, adjuvant treatment, and mutational tumor status (*KRAS* or *EGFR*), was collected from medical records. Follow-up information for recurrence-free survival (RFS) and OS rates were also retrieved from the patients’ electronic medical records (Supplementary Table 7).

### mIF staining and analysis

mIF staining was performed using methods similar to those previously described and validated^16^. Briefly, formalin-fixed, paraffin-embedded TMA sections of 4-µm thickness were stained using 5 panels containing the following antibodies: panel 1, cytokeratin (CK), CD3, CD8, PD-1, PD-L1, and CD68; panel 2, CK, CD3, CD8, CD45RO, granzyme B (GZB), and FOXP3; panel 3, CK, CD3, PD-L1, B7-H3, B7-H4, IDO-1, and VISTA; panel 4, CK, CD3, ICOS, LAG3, OX40, TIM3, and CD20; and panel 5, CK, Arg-1, CD11b, CD14, CD33, CD66b, and CD68. All markers were stained in sequence using their respective fluorophore contained in the Opal 7 IHCkit (catalog #NEL797001KT; Akoya Biosciences, Marlborough, MA) for the panels with 6 antibodies, and coumarin fluorophore (catalog #NEL703001KT; Akoya Biosciences) was added in the panels with 7 antibodies (Supplementary Table 8). Positive (human reactive tonsils) and negative or autofluorescence controls (human reactive tonsils including the antibodies but without any fluorophores) were included in each run of staining^46^. Supplementary Fig. 3 shows representative individual marker expressions from the TMA across the panels. The stained slides were scanned using the multispectral microscope Vectra Polaris 1.0.13 imaging system (Akoya Biosciences) under fluorescence conditions at low magnification (10×), and then each core was viewed at high magnification (20×). Each core from the TMAs was analyzed using the InForm 2.4.0 digital image analysis software (Akoya Biosciences). Marker co-localization was used to identify the most relevant specific cell phenotypes from each mIF panel, as shown in Supplementary Table 1. Densities of each cell phenotype were quantified, and the final data were expressed as the number of cells/mm^2^. Experiments and quantifications related to the presented results were conducted once. The data were consolidated using R studio 3.5.3 (Phenopter 0.2.2 packet; https://rdrr.io/github/akoyabio/phenoptrReports/f/, Akoya Biosciences).

### Immune cell phenotype characterization

We created cord plots to visualize cell phenotypes interaction based on the co-expression of markers using the markers from each mIF panel. Additionally, dimensional reduction was applied using uniform manifold approximation and projection [UMAP, (https://github.com/lmcinnes/umap)] to visualize all possible cell phenotypes observed in each panel using the tumor cores^47,48^. The results were plotted using R studio software v.3.6.1 and Python v. 3.8.9.

### Spatial cellular distribution analysis

To define spatial pattern distributions and cellular interactions between CK + malignant cells and TAICs, we compared the empirically derived cross G function curve (https://research.csiro.au/software/r-workshop-notes) with the theoretical Poisson curve (median distances of the specific cells from malignant cells between samples), obtained by assuming the same intensity (absolute number of cells) pattern is observed in each sample^49^, to characterize patterns of cellular distribution and possible interactions between the cells. A total of 26 cell phenotypes, including T-cells, B-cells, granulocytes, myeloid cells, and macrophages, were studied using this approach, excluding the cell phenotypes with <2 cells/mm2 as the median value (Table 1) to avoid any biases in the analysis. Furthermore, using the spatial point pattern distribution of the cell phenotypes relative to malignant cells, we measured the distance from CK + malignant cells to the 26 cell phenotypes mentioned above (Table 1) and from CK + PD-L1 + , CK + PD-L1^neg^, CK + B7-H3 + , CK + B7-H4 + , CK + IDO-1+ and CD3 + CD8^neg^FOXP3 + to different cell phenotype included in the panels (Supplementary Table 3) using a matrix created with each cell’s X and Y coordinates in R studio software v.3.6.1. A total of 128 distances were measure. We applied the median nearest neighbor function (Phenopter 0.2.2 packet; https://rdrr.io/github/akoyabio/phenoptrReports/f/, Akoya Biosciences) from CK + malignant cells to CD3 + T-cells, CD20 + B-cells, CD68 + macrophages, and CD66b + granulocytic cells (PMNs), as well as to the other cell phenotypes, to determine where these TAICs were located; specifically, whether the TAICs were close to (equal to or less than the median distance) or far from (more than the median distance) the CK + malignant cells^47^.

We expanded the characterization of cellular distribution patterns and distances, combining the results of the leading 26 cell phenotypes to identify four groups of cellular immunologic distribution from these cell phenotypes:

Group 1. Mixed pattern with close median distances to the malignant cells

Group 2. Mixed pattern with long median distances to the malignant cells

Group 3. Unmixed pattern with close median distances to the malignant cells

Group 4. Unmixed pattern with long median distances to the malignant cells

Finally, using CD3 + T-cells as a predominant cell population in the tumor samples, we identified four groups of cells and their associated cell phenotype densities to characterize possible differences in tumor microenvironments.

### Statistical methods

Because our principal focus was not to measure cellular phenotype densities, the densities and distances of various cell phenotypes from malignant cells were dichotomized: values greater than the median were considered high density or long distance and values equal to or lower than the median were considered low density or close distance. For patterns of cellular distribution, a score ranging from −10 to 10 in the comparison of the G function curve with the theoretical Poisson curve indicated a mixed pattern and a score >10 indicated an unmixed pattern, for various TAICs. Nonparametric tests were used to assess associations in the patterns of cellular distribution (26 cell phenotypes) and spatial distance analysis from malignant cells to TAICs (128 distances), and associations between cellular distribution patterns and clinicopathologic features were evaluated using the Wilcoxon rank-sum or Kruskal-Wallis test. For univariate analyses, only the most abundant cell phenotypes (>2 cells/mm^2^) were used, and the Kaplan-Meier method and log-rank test were used to determine whether patterns of cellular distribution, cellular distances, or cellular immunologic distribution groups were associated with RFS or OS. Additionally, Cox proportional hazards models were used to evaluate associations between cellular distribution, cellular distances, and the four immunologic cellular distribution groups, controlling for clinicopathologic characteristics. Un-adjusted *P*-value of less than 0.05 was considered statistically significant. All analyses and data visualization were performed in R 3.6.0 and 3.6.1 (released April 2019; https://www.r-project.org), R studio 3.5.3 (Phenopter 0.2.2 packet; https://rdrr.io/github/akoyabio/phenoptrReports/f/, Akoya Biosciences), Python v.3.8.9, and/or GraphPad Prism v.9.0.0.

### Reporting summary

Further information on research design is available in the Nature Portfolio Reporting Summary linked to this article.

## Supplementary information

Supplementary Information

Reporting Summary

## Data Availability

The authors declare that the data supporting the findings of this study are available within the manuscript and its supplementary information files. The data is provided as a source data file. Other relevant de-identified data images related to the current study are available in the repository, https://bitbucket.org/chuymtz/tma3/src/master/ from the corresponding author (E.R.P) upon academic request and will require the researcher to sign a data access agreement with the University of Texas MD Anderson Cancer Center after approval. Source data are provided with this paper.

## Source data

Source Data
