# Supplementary material for: Immune cellular patterns of distribution affect outcomes of patients with non-small cell lung cancer
Source: Nat Commun. 2023 Apr 25;14:2364. doi: 10.1038/s41467-023-37905-y (PMC10130161; doi:10.1038/s41467-023-37905-y)
Supplement: Supplementary file 1 — Supplementary Information [file 41467_2023_37905_MOESM1_ESM.pdf]

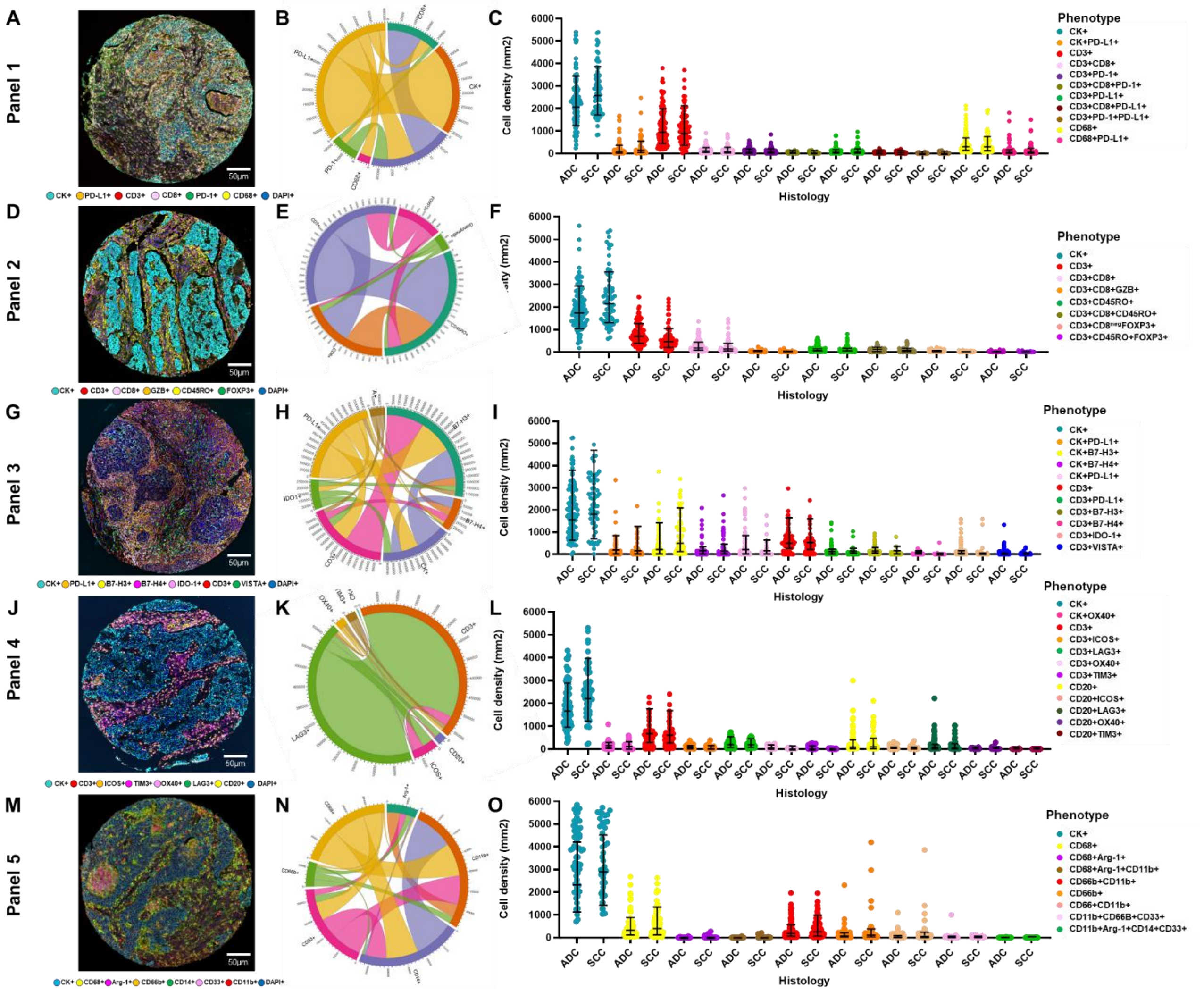

Supplementary Figure 1. Representative examples of multispectral images and individual cord plots for each multiplex immunofluorescence (mIF) panel and the most commonly observed cell phenotype densities in each panel. Composite spectral mixing images from mIF (20× magnification, scale bars represent 50μm on each image) showing the diversity of marker co-expression for (A) panel 1, (D) panel 2, (G) panel 3, (J) panel 4, and (M) panel 5. (B, E, H, K, N) Chord diagram visualizations showing the diversity of overall inter-relationships between co-expression of markers in each mIF panel. (C, F, I, L, O) Scatter plots of individual densities and their error bars presented as median values from the most common cell phenotype densities observed in the mIF panels, divided by histologic type (adenocarcinoma [ADC=142 samples] or squamous cell carcinoma [SCC=83 samples]). Panel 1, 11 cells phenotypes; panel 2, 8 cell phenotypes; panel 3, 11 cell phenotypes; panel 4, 12 cell phenotypes; panel 5, 9 cell phenotypes. Experiments and quantifications related to the presented results were conducted once. The images were generated using Vectra-Polaris 1.0.13 scanner system and InForm 2.4.8 image analysis software (Akoya Biosciences), chord diagrams were generated using R studio software version 3.6.1, and scatter plots and error bars were generated using GraphPad Prism v.9.0.0. Data from 225 samples was used in mIF. (Source data is provided as a source data file).

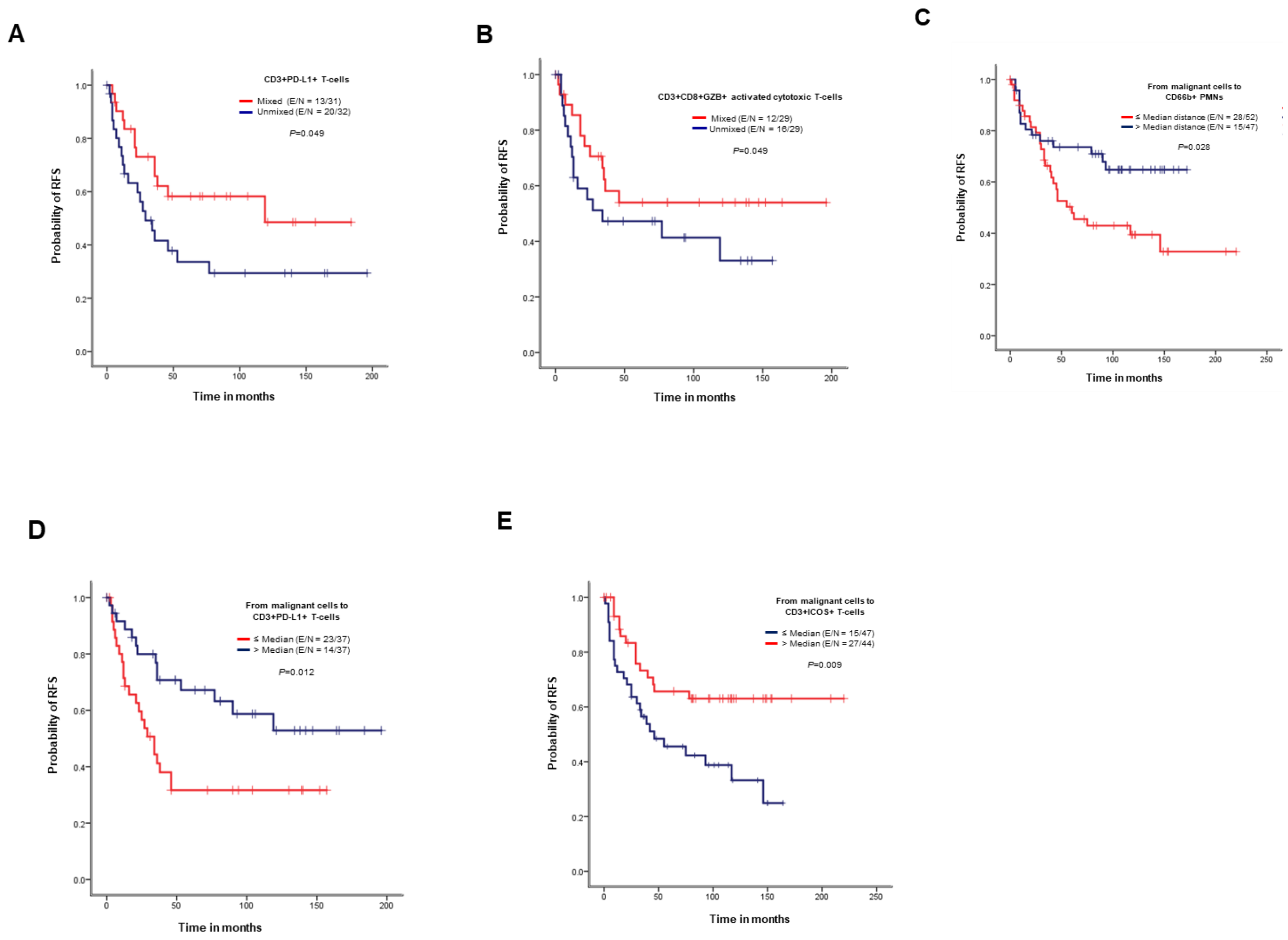

Supplementary Figure 2. Kaplan-Meier analysis of recurrence-free survival (RFS) by patterns of distribution and distance from malignant cells to various immune cell subpopulations. Blue lines indicate an unmixed pattern and red lines indicate a mixed pattern between malignant cells and immune cells for (A) CD3+PD-L1+ T-cells and (B) CD3+CD8+granzyme B (GZB)+ activated cytotoxic T-cells in lung squamous cell carcinoma specimens. Blue lines indicate long (>median) distances and red lines indicate close (≤median) distances from malignant cells to (C) CD66b+ granulocytes (PMNs) in lung adenocarcinoma specimens and (D) CD3+PD-L1+ T-cells and (E) CD3+ICOS+ T-cells in lung squamous cell carcinoma specimens. Kaplan-Meier curves and log-rank test were used and generated by the R studio software version 3.6.0. using the distribution patterns and distances from the 26-cell phenotypes with un-adjusted *P*-values (n=225 samples), (Source data is provided as a source data file).

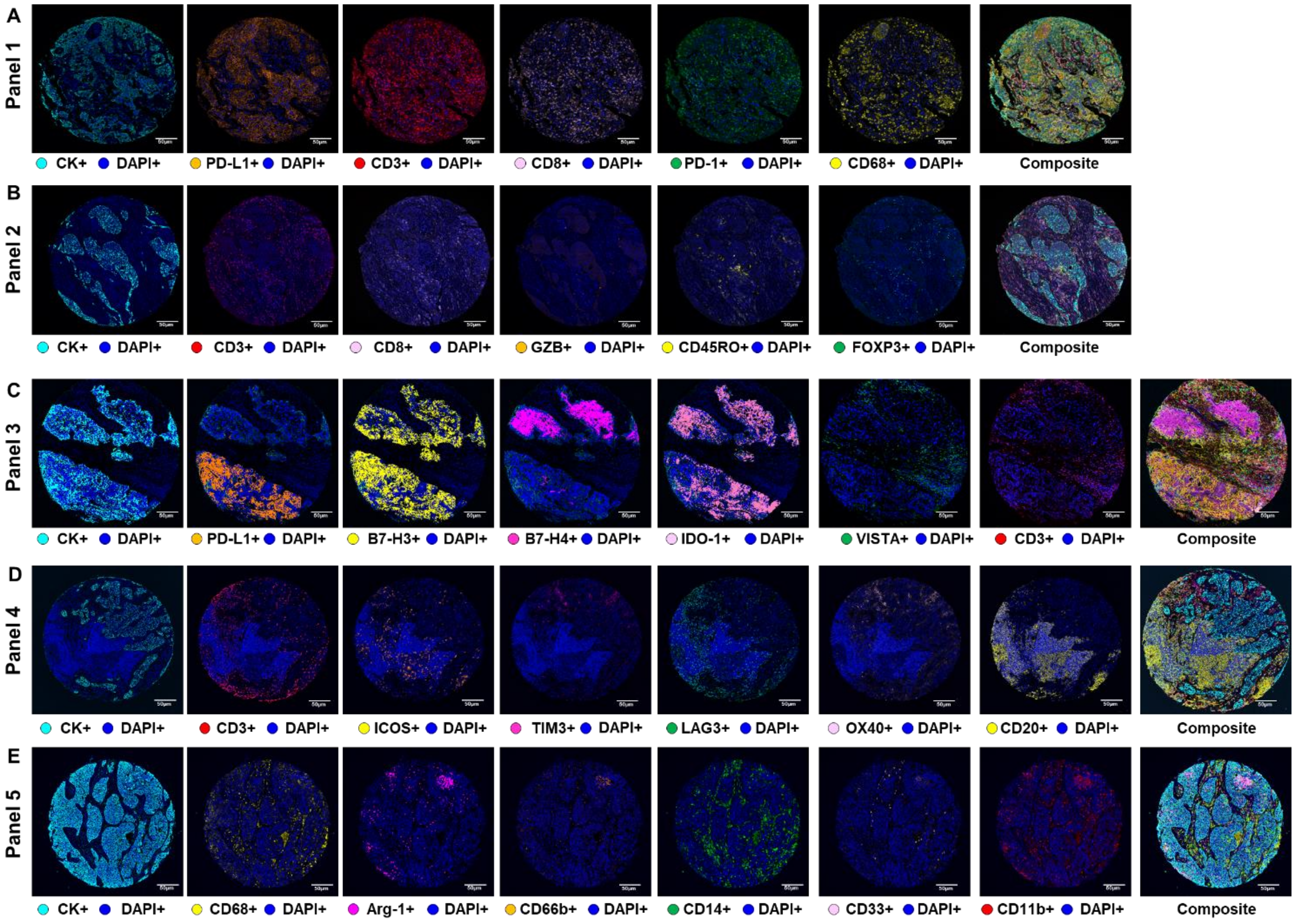

Supplementary Figure 3. Representative examples of unmixed and mixed multispectral image samples from the five-multiplex immunofluorescence (mIF) panels. Unmixed images (20× magnification, scale bars represent 50μm on each image) showing the individual marker's expression plus DAPI (4′,6-diamidino-2-phenylindole) and their composite mixed image contained all the markers plus DAPI from the mIF panel 1, cytokeratin (CK), CD3, CD8, PD-1, PD-L1, and CD68 (A); panel 2, CK, CD3, CD8, CD45RO, granzyme B (GZB), and FOXP3 (B); panel 3, CK, CD3, PD-L1, B7-H3, B7-H4, IDO-1, and VISTA (C); panel 4, CK, CD3, ICOS, LAG3, OX40, TIM3, and CD20 (D); and panel 5, CK, Arg-1, CD11b, CD14, CD33, CD66b, and CD68 (E). Experiments and quantifications related to the presented results were conducted once. The images were generated using the Vectra-Polaris 1.0.13 scanner system and InForm 2.4.8 image analysis software (Akoya Biosciences). Data from 225 samples was used in mIF. (Source data is provided as a source data file).

Supplementary Table 1. Frequently observed phenotypes in the five multiplex immunofluorescence panels in the non-small cell lung cancer cohort (n = 225).

Panel	Marker co-expression	Phenotype
1	CK+	All malignant cells
	CK+PD-L1+	All malignant cells expressing PD-L1
	CD3+	All T lymphocytes
	CD3+CD8+	Cytotoxic T-cells
	CD3+PD-1+	Antigen-experienced T-cells
	CD3+CD8+PD-1+	Antigen-experienced cytotoxic T-cells
	CD3+CD8+PD-L1+	Cytotoxic T-cells expressing PD-L1
	CD3+PD-1+PD-L1+	Antigen-experienced PD-L1 T-cells
	CD3+CD8+PD-1+PD-L1+	Antigen-experienced PD-L1 cytotoxic T-cells
	CD68+	All tumor-associated macrophages
	CD68+PD-L1+	Tumor-associated macrophages expressing PD-L1
2	CK+	All malignant cells
	CD3+	All T lymphocytes
	CD3+CD8+	Cytotoxic T-cells
	CD3+CD8+GZB+	Activated cytotoxic T-cells
	CD3+CD45RO+	Memory T-cells
	CD3+CD8+CD45RO+	Effector/memory T-cells
	CD3+CD8^{neg}FOXP3+	Regulatory T-cells
	CD3+CD45RO+FOXP3+	Regulatory/memory T-cells
3	CK+	All malignant cells
	CK+PD-L1+	Malignant cells expressing PD-L1
	CK+B7-H3+	Malignant cells expressing B7-H3
	CK+B7-H4+	Malignant cells expressing B7-H4
	CK+IDO-1+	Malignant cells expressing IDO-1
	CD3+	All T lymphocytes
	CD3+PD-L1+	T-cells expressing PD-L1
	CD3+B7-H3+	T-cells expressing B7-H3
	CD3+B7-H4+	T-cells expressing B7-H4
	CD3+IDO-1+	T-cells expressing IDO-1
	CD3+VISTA+	T-cells expressing VISTA
4	CK+	All malignant cells
	CK+OX40+	Malignant cells expressing OX40
	CD3+	All T lymphocytes
	CD3+ICOS+	T-cells expressing ICOS
	CD3+LAG3+	T-cells expressing LAG3
	CD3+OX40+	T-cells expressing OX40
	CD3+TIM3+	T-cells expressing TIM3
	CD20+	All B lymphocytes
	CD20+ICOS+	B-cells expressing ICOS
	CD20+LAG3+	B-cells expressing LAG3
	CD20+OX40+	B-cells expressing OX40
	CD20+TIM3+	B-cells expressing TIM3
5	CK+	All malignant cells
	CD68+	All tumor-associated macrophages
	CD68+Arg-1+	Type II tumor-associated macrophages
	CD68+Arg-1+CD11b+	Myeloid type II tumor-associated macrophages
	CD68+CD11b+	Dendritic macrophages
	CD66b+	All granulocytes
	CD11b+CD66b+	Myeloid granulocytes
	CD11b+Arg-1+CD14+CD33+	Monocytic myeloid-derived suppressor cells
	CD11b+CD66b+CD33+	Granulocytic myeloid-derived suppressor cells

Note: CK, cytokeratin; GZB, granzyme B.

Supplementary Table 2. Median densities of cell phenotypes by histologic type.

Panel	Phenotype	Cells/mm^2			
		NSCLC (%)*	Adenocarcinoma (%)*	Squamous cell carcinoma (%)*	*P*†
1	CK+	2342.70	2281.72	2609.02	0.005
	CK+PD-L1+	90.63 (5.51)	87.74 (4.50)	133.65 (4.87)	0.051
	CD3+	834.54	1012.85	1022.33	0.910
	CD3+CD8+	128.42	199.75	143.16	0.043
	CD3+PD-1+	39.53	53.32	71.69	0.607
	CD3+PD-L1+	45.15	47.59	68.09	0.046
	CD3+CD8+PD-1+	4.75	8.73	6.27	0.479
	CD3+CD8+PD-L1+	6.42	8.26	13.12	0.139
	CD3+PD-1+PD-L1+	2.07	2.94	4.23	0.055
	CD68+	277.14	312.32	382.11	0.963
	CD68+PD-L1+	40.48	39.47	58.26	0.259
2	CK+	1911.12	1812.05	2232.66	0.013
	CD3+	961.09	1094.53	1066.96	0.123
	CD3+CD8+	144.60	193.12	136.25	0.024
	CD3+CD8+GZB+	5.31	6.00	6.93	0.789
	CD3+CD45RO+	57.39	93.95	59.29	0.008
	CD3+CD8+CD45RO+	25.44	51.91	28.06	0.037
	CD3+CD8^{neg}FOXP3+	24.10	30.02	25.51	0.328
	CD3+CD45RO+FOXP3+	3.67	5.26	4.73	0.109
3	CK+	2013.57	1797.32	2307.99	0.050
	CK+PD-L1+	88.90 (4.41)	91.40 (5.08)	140.72 (6.09)	0.089
	CK+B7-H3+	307.79 (15.29)	172.92 (9.59)	745.80 (32.31)	<0.001
	CK+B7-H4+	7.08 (0.35)	1.05 (0.05)	31.78 (1.37)	<0.001
	CK+IDO-1+	19.17 (0.95)	34.06 (1.89)	5.09 (0.22)	0.015
	CD3+	813.77	974.78	948.77	0.155
	CD3+PD-L1+	38.72	32.21	50.76	0.044
	CD3+B7-H3+	8.86	12.73	6.32	0.388
	CD3+B7-H4+	0.00	0.00	0.00	0.267
	CD3+IDO-1+	1.50	5.15	0.47	0.005
	CD3+VISTA+	1.76	2.01	1.63	0.540
4	CK+	2028.33	1743.00	2428.43	0.001
	CK+OX40+	25.38 (1.25)	29.97 (1.72)	19.33 (0.79)	0.557
	CD3+	929.81	1040.91	913.43	0.143
	CD3+ICOS+	36.19	36.92	33.42	0.841
	CD3+LAG3+	229.81	240.34	213.43	0.651
	CD3+OX40+	12.07	14.68	8.27	0.134
	CD3+TIM3+	6.64	7.90	4.20	0.020
	CD20+	80.07	73.82	97.42	0.657
	CD20+ICOS+	7.44	7.17	7.87	0.497
	CD20+OX40+	4.72	6.79	2.76	0.157
	CD20+TIM3+	0.36	0.42	0.00	0.072
	CD20+LAG3+	31.10	28.62	35.20	0.922
5	CK+	2528.17	2145.2	2617.60	0.004
	CD68+	359.51	300.73	495.39	0.064
	CD68+Arg-1+	1.23	1.17	1.95	0.250
	CD68+Arg-1+CD11b+	1.20	1.17	1.73	0.314
	CD68+CD11b+	231.11	180.61	344.74	0.012
	CD66b+	83.79	70.32	111.89	0.045
	CD11b+CD66b+	33.58	29.43	41.58	0.018
	CD11b+Arg-1+CD14+CD33+	1.04	0.81	1.11	0.765
	CD11b+CD66b+CD33+	11.44	9.57	13.74	0.181

Note: NSCLC, non-small cell lung cancer; CK, cytokeratin; GZB, granzyme B.

*Percentage of immune checkpoints related to total CK+ malignant cells.

† P values indicate comparison between adenocarcinoma and squamous cell carcinoma using Kruskal-Wallis test and un-adjusted P-values.

Supplementary Table 3. Median distances from malignant cells, T-cells, and macrophages to various cell phenotypes.

		Median distance, µm			
		From CK+PD-L1+		From CK+PD-L1^{neg}	
**Panel**	**Phenotype**				
1	CD3+	34.15		16.19	
	CD3+CD8+	76.03		45.80	
	CD3+PD-1+	113.35		68.20	
	CD3+PD-L1+	217.38		166.86	
	CD3+CD8+PD-1+	115.38		69.65	
	CD3+CD8+PD-L1+	218.40		147.99	
	CD3+PD-1+PD-L1+	338.72		264.19	
	CD68+	42.31		12.90	
	CD68+PD-L1+	119.71		51.89	
	CD68+PD-L1^{neg}	45.18		14.39	
		**From CD3+CD8^{neg}FOXP3+**			
2	CD3+	8.32			
	CD3+CD8+	26.73			
	CD3+CD8+GZB+	184.98			
	CD3+CD45RO+	40.83			
	CD3+CD8+CD45RO+	43.05			
	CD3+CD45RO+FOXP3+	68.98			
		**From CK+PD-L1+**	**From CK+B7-H3+**	**From CK+B7-H4+**	**From CK+IDO-1+**
3	CD3+	25.63	27.54	24.16	18.51
	CD3+B7-H3+	37.96	65.64	75.92	53.51
	CD3+B7-H4+	58.69	41.27	82.03	43.36
	CD3+IDO-1+	181.53	234.05	36.65	207.07
	CD3+PD-L1+	60.66	62.59	26.67	24.07
	CD3+VISTA+	81.70	82.81	88.93	65.85

Note: The table shows fifty distances measurements from malignant cells expressing immune checkpoint markers, malignant cells negative to PD-L1 expression, and regulatory T-cells to twenty-two T-cell phenotypes that were analyzed and associated with clinicopathologic features.

Supplementary Table 4. Cox proportional hazards regression model of overall survival in patients with non-small cell lung cancer for the mixed compared with unmixed pattern relative to malignant cells, adjusted for clinicopathologic variables.

**Variable**	**B**	**SE**	**Wald**	**HR**	**95% CI for Exp(B)**	***P****
Histologic type (ADC vs SCC)	0.093	0.338	0.077	1.098	0.566-2.129	0.782
Smoker (no vs yes)	-1.758	0.931	3.566	0.172	0.028-1.069	0.059
Tumor size (≤1.5 cm vs >3.8 cm)	-1.048	0.335	9.803	0.351	0.182-0.676	**0.002**
KRAS (wild-type vs mutant)	1.326	0.522	6.447	3.765	1.353-10.478	**0.011**
EGFR (wild-type vs mutant)	-0.443	0.698	0.404	0.642	0.164-2.519	0.525
Mixed vs unmixed pattern						
CD3+	0.379	0.471	0.646	1.461	0.580-3.680	0.421
CD3+CD8+	-1.939	1.707	1.290	0.144	0.005-4.084	0.256
CD3+CD8+GZB+	-0.149	1.122	0.018	0.862	0.096-7.771	0.894
CD3+CD45RO+	-0.214	1.158	0.034	0.807	0.084-7.805	0.853
CD3+CD8+CD45RO+	0.618	0.806	0.587	1.854	0.382-9.001	0.444
CD3+CD8^{neg}FOXP3+	0.448	1.310	0.117	1.565	0.120-20.416	0.733
CD3+CD45RO+FOXP3+	0.377	0.351	1.159	1.459	0.734-2.899	0.282
CD3+PD1+	0.464	0.453	1.049	1.590	0.655-3.860	0.306
CD3+CD8+PD1+	0.383	0.400	0.919	1.467	0.670-3.213	0.338
CD3+PD-L1+	-0.613	0.514	1.424	0.542	0.198-1.483	0.233
CD3+CD8+PD-L1	-0.092	0.439	0.044	0.912	0.386-2.155	0.833
CD3+PD1+PD-L1+	0.353	0.470	0.565	1.424	0.567-3.579	0.452
CD3+B7H3+	-1.978	2.308	0.734	0.138	0.002-12.753	0.391
CD3+OX40+	-5.213	2.705	3.712	0.005	0.000-1.094	0.054
CD3+TIM3+	-7.949	4.033	3.885	0.000	0.000-0.957	**0.049**
CD3+LAG3+	-3.491	3.423	1.040	0.030	0.000-4.988	0.308
CD3+ICOS+	-3.218	5.560	0.335	0.040	0.000-3.922	0.563
CD20+	6.727	4.259	2.495	834.726	0.198-8.514	0.114
CD20+ICOS+	1.770	1.595	1.231	5.871	0.258-3.758	0.267
CD20+LAG3+	-7.539	4.015	3.526	0.001	0.000-1.390	0.060
CD20+OX40+	5.582	5.903	0.894	265.722	0.003-3.930	0.344
CD68+	0.503	0.437	1.328	1.654	0.703-3.891	0.249
CD68+PD-L1	-0.189	0.323	0.341	0.828	0.440-1.559	0.559
CD68+CD11b+	-1.760	1.489	1.396	0.172	0.009-3.187	0.237
CD66b+	1.326	3.184	0.173	3.764	0.007-9.897	0.677
CD66b+CD11b+	4.208	5.749	0.536	67.202	0.001-7.698	0.464
CD33+CD66b+CD11b+	-2.732	5.289	0.267	0.065	0.000-68.717	0.606

Note: B, unstandardized regression weight; SE, multiple linear regression; Wald, Wald test; HR, hazard ratio; CI, confidence interval; ADC, adenocarcinoma; SCC, squamous cell carcinoma; GZB, granzyme B.

*Boldface indicates statistically significant difference using Cox proportional-hazards model with un-adjusted P-values for clinicopathologic variables. The table shows the analysis of spatial distribution patterns of the 27 most relevant cell phenotypes from the different multiplex immunofluorescence panels adjusted by clinicopathologic features.

Supplementary Table 5. Cox proportional hazards regression model of overall survival in patients with non-small cell lung cancer for close compared with long distances from malignant cells, adjusted for clinicopathologic variables.

**Variable**	**B**	**SE**	**Wald**	**HR**	**95% CI for Exp(B)**	***P******
Histologic type (ADC vs SCC)	-8.882	3.643	5.943	0.000	0.000-0.175	**0.015**
Smoker (no vs yes)	40.279	15.617	6.652	311.000	158.436-610.000	**0.010**
Tumor size (≤1.5 cm vs >3.8 cm)	-15.224	6.396	5.666	0.000	0.000-0.068	**0.017**
KRAS (wild-type vs mutant)	-15.552	6.252	6.188	0.000	0.000-0.037	**0.013**
EGFR (wild-type vs mutant)	50.367	14.488	12.087	748.000	348.000-160.000	**0.001**
Close vs long distance from malignant cells						
CD3+	-36.110	9.640	14.031	0.000	0.000-0.000	**0.000**
CD3+CD8+	-13.262	7.813	2.881	0.000	0.000-7.779	0.090
CD3+CD8+GZB+	-13.288	5.617	5.596	0.000	0.000-0.102	**0.018**
CD3+CD45RO+	-4.676	5.772	0.656	0.009	0.000-763.242	0.418
CD3+CD8+CD45RO+	-18.353	7.079	6.721	0.000	0.000-0.011	**0.010**
CD3+CD8^{neg}FOXP3+	16.908	5.572	9.209	220.080	398.512-121.000	**0.002**
CD3+CD45RO+FOXP3+	62.550	20.627	9.196	146.000	405.000-580.000	**0.002**
CD3+PD-1+	-33.343	10.893	9.369	0.000	0.000-0.000	**0.002**
CD3+CD8+PD-1+	1.174	3.545	0.110	3.233	0.003-336.778	0.741
CD3+PD-L1+	-27.268	8.001	11.616	0.000	0.000-0.000	**0.001**
CD3+CD8+PD-L1+	25.574	8.672	8.698	127.000	531.863-3074.000	**0.003**
CD3+PD-1+PD-L1+	7.959	4.906	2.632	2860.982	0.191-428.450	0.105
CD3+B7H3+	-1.115	1.959	0.324	0.328	0.007-15.250	0.569
CD3+OX40+	8.824	3.122	7.988	95.453	14.951-30.350	**0.005**
CD3+TIM3+	-9.908	3.224	9.446	0.000	0.000-0.028	**0.002**
CD3+LAG3+	26.392	8.144	10.502	289.000	338.821-247.000	**0.001**
CD3+ICOS+	-4.402	2.149	4.194	0.012	0.000-0.828	**0.041**
CD20+	-13.896	6.835	4.134	0.000	0.000-0.606	**0.042**
CD20+ICOS+	-2.166	1.625	1.777	0.115	0.005-2.768	0.182
CD20+LAG3+	12.022	7.416	2.628	166.529	0.081-341.000	0.105
CD20+OX40+	0.583	1.713	0.116	1.791	0.062-51.386	0.734
CD68+	-2.416	3.874	0.389	0.089	0.000-177.076	0.533
CD68+PD-L1+	25.233	8.097	9.710	908.000	116.066-709.000	**0.002**
CD68+CD11b+	-7.649	3.971	3.710	0.000	0.000-1.144	0.054
CD66b+CD11b+	-14.960	5.787	6.683	0.000	0.000-0.027	**0.010**
CD66b+	2.988	2.657	1.265	19.852	0.109-362.598	0.261
CD11b+CD66b+CD33+	2.139	2.194	0.950	8.487	0.115-625.386	0.330

Note: B, unstandardized regression weight; SE, multiple linear regression; Wald, Wald test; HR, hazard ratio; CI, confidence interval; ADC, adenocarcinoma; SCC, squamous cell carcinoma, GZB, granzyme B.

*Boldface indicates statistically significant difference using Cox proportional-hazards model with un-adjusted *P*-values for clinicopathologic variables.

The table shows the analysis of distances from malignant cells to the 27 most relevant cell phenotypes of the multiplex immunofluorescence panels adjusted by clinicopathologic features.

Supplementary Table 6. Cox proportional hazards regression model of overall survival in patients with non-small cell lung cancer for the four groups of cellular immunologic patterns, adjusted for clinicopathologic variables.

Variable	B	SE	Wald	HR	95% CI for Exp(B)	*P**
Histologic type (ADC vs SCC)	0.800	1.237	0.419	2.226	0.197-25.152	0.518
Smoker (no vs yes)	-7.812	3.939	3.934	0.000	0.000-0.912	**0.047**
Tumor size (≤1.5 cm vs >3.8 cm)	-1.165	0.797	2.134	0.312	0.065-1.489	0.144
KRAS (wild-type vs mutant)	0.532	0.953	0.311	1.701	0.263-11.006	0.577
EGFR (wild-type vs mutant)	-6.622	2.922	5.135	0.001	0.000-0.409	**0.023**
CD3+ (group 1 vs group 4)	2.565	3.090	0.689	13.001	0.030-5543.724	0.406
CD3+ (group 2 vs group 4)	-2.010	1.699	1.399	0.134	0.005-3.745	0.237
CD3+ (group 3 vs group 4)	-11.314	5.418	4.361	0.000	0.000-0.499	**0.037**
CD3+CD8+ (group 1 vs group 4)	-7.522	4.271	3.102	0.001	0.000-2.337	0.078
CD3+CD8+ (group 2 vs group 4)	-1.446	1.367	1.118	0.236	0.016-3.436	0.290
CD3+CD8+GZB+ (group 1 vs group 4)	-1.049	2.601	0.163	0.350	0.002-57.275	0.687
CD3+CD8+GZB+ (group 2 vs group 4)	-0.456	2.516	0.033	0.634	0.005-87.813	0.856
CD3+CD45RO+ (group 1 vs group 4)	-12.135	8.891	1.863	0.000	0.000-198.621	0.172
CD3+CD45RO+ (group 2 vs group 4)	-0.234	1.373	0.029	0.792	0.054-11.678	0.865
CD3+CD45RO+ (group 3 vs group 4)	-2.076	2.998	0.480	0.125	0.000-44.665	0.489
CD3+CD8+CD45RO+ (group vs group 4)	1.917	2.048	0.877	6.801	0.123-376.288	0.349
CD3+CD8+CD45RO+ (group 2 vs group 4)	-2.601	1.156	5.059	0.074	0.008-0.716	**0.024**
CD3+CD8^{neg}FOXP3+ (group 2 vs group 4)	8.510	3.160	7.254	496.458	10.148-242.835	**0.007**
CD3+CD8^{neg}FOXP3+ (group 3 vs group 4)	6.987	3.434	4.140	108.501	1.292-906.134	**0.042**
CD3+CD45RO+FOXP3+ (group 1 vs group 4)	-0.161	1.639	0.010	0.851	0.034-21.121	0.922
CD3+CD45RO+FOXP3+ (group 2 vs group 4)	1.791	1.090	2.700	5.998	0.708-50.827	0.100
CD3+CD45RO+FOXP3+ (group 3 vs group 4)	2.011	1.157	3.023	7.470	0.774-72.069	0.082
CD3+PD-1+ (group 1 vs group 4)	3.854	3.138	1.508	47.173	0.101-221.041	0.219
CD3+PD-1+ (group 2 vs group 4)	1.764	1.816	0.943	5.835	0.166-205.211	0.332
CD3+PD-1+ (group 3 vs group 4)	-0.811	2.500	0.105	0.445	0.003-59.739	0.746
CD3+CD8+PD-1+ (group 1 vs group 4)	-1.759	1.687	1.087	0.172	0.006-4.699	0.297
CD3+CD8+PD-1+ (group 2 vs group 4)	-1.008	1.565	0.415	0.365	0.017-7.831	0.519
CD3+CD8+PD-1+ (group 3 vs group 4)	0.804	1.646	0.239	2.236	0.089-56.265	0.625
CD3+PD-L1+ (group 1 vs group 4)	-2.611	2.203	1.405	0.073	0.001-5.510	0.236
CD3+PD-L1+ (group 2 vs group 4)	-1.766	1.238	2.034	0.171	0.015-1.937	0.154
CD3+PD-L1+ (group 3 vs group 4)	3.337	3.555	0.881	28.147	0.026-299.325	0.348
CD3+CD8+PD-L1+ (group 1 vs group 4)	0.945	1.906	0.246	2.573	0.061-107.755	0.620
CD3+CD8+PD-L1+ (group 2 vs group 4)	2.448	1.778	1.897	11.570	0.355-377.126	0.168
CD3+CD8+PD-L1+ (group 3 vs group 4)	0.928	1.653	0.315	2.529	0.099-64.603	0.575
CD3+PD-1+PD-L1+ (group 1 vs group 4)	3.461	1.949	3.153	31.837	0.698-145.264	0.076
CD3+PD-1+PD-L1+ (group 2 vs group 4)	0.785	1.556	0.254	2.192	0.104-46.251	0.614
CD3+PD-1+PD-L1+ (group 3 vs group 4)	3.071	1.504	4.169	21.557	1.131-410.864	**0.041**
CD3+B7-H3+ (group 1 vs group 4)	0.000	11.204	0.000	1.000	0.000-891.000	1.000
CD3+B7-H3+ (group 2 vs group 4)	-22.419	75.405	0.088	0.000	0.000-2.810	0.766
CD3+OX40+ (group 1 vs group 4)	-20.472	19.313	1.124	0.000	0.000-353.570	0.289
CD3+OX40+ (group 2 vs group 4)	-4.087	7.654	0.285	0.017	0.000-549.984	0.593
CD3+OX40+ (group 3 vs group 4)	-16.344	13.628	1.438	0.000	0.000-317.771	0.230
CD68+ (group 1 vs group 4)	-1.130	1.582	0.510	0.323	0.015-7.181	0.475
CD68+ (group 2 vs group 4)	-0.597	.838	0.507	0.551	0.106-2.848	0.477
CD68+ (group 3 vs group 4)	-2.618	2.122	1.522	0.073	0.001-4.669	0.217
CD68+PD-L1+ (group 1 vs group 4)	-0.122	1.213	0.010	0.885	0.082-9.531	0.920
CD68+PD-L1+ (group 2 vs group 4)	0.421	1.125	0.140	1.523	0.168-13.806	0.708
CD68+PD-L1+ (group 3 vs group 4)	-0.467	1.491	0.098	0.627	0.034-11.657	0.754

Note: HR, hazard ratio; CI, confidence interval; ADC, adenocarcinoma; SCC, squamous cell carcinoma; GZB, granzyme B; group 1, mixed pattern with close median distances to malignant cells; group 2, mixed pattern with long median distances to malignant cells; group 3, unmixed pattern with close median distances to malignant cells; group 4, unmixed pattern with long median distances to malignant cells.

*Boldface indicates statistically significant difference using Cox proportional-hazards model with un-adjusted *P*-values for clinicopathologic variables. The table shows the analysis of cellular immunologic patterns of 16 relevant cell phenotypes of the multiplex immunofluorescence panels adjusted by clinicopathologic features.

Supplementary Table 7. Characteristics of the 225 patients with NSCLC whose tumor specimens were included in our study.

		Type of NSCLC, no. (%)	
**Characteristic**	**All patients, n=225**	**Adenocarcinoma, n=142**	**Squamous cell carcinoma, n=83**
Median age	66 years	64.5 years	67 years
Sex			
Female	107 (48)	71 (50)	36 (43)
Male	118 (52)	71 (50)	47 (57)
Tobacco history			
No	18 (8)	17 (12)	1 (1)
Yes	207 (92)	125 (88)	82 (99)
Smoking status			
Never	18 (8)	17 (12)	1 (1)
Former	102 (45)	64 (45)	38 (46)
Current	105 (47)	61 (43)	44 (53)
Median tumor size	3.5 cm	3.15 cm	3.8 cm
Tumor status*			
T_1	70 (31)	43 (30)	27 (33)
T_2	90 (40)	65 (46)	25 (30)
T_3	40 (18)	21 (15)	19 (23)
T_4	25 (11)	13 (9)	12 (14)
Nodal status*			
N_0	148 (66)	98 (69)	50 (60)
N_1	46 (20)	25 (18)	21 (25)
N_2	31 (14)	19 (13)	12 (15)
Stage*			
I	102 (45)	71 (50)	31 (37)
II	60 (27)	35 (25)	25 (30)
III	63 (28)	36 (25)	27 (33)
Adjuvant therapy†			
No	134 (60)	86 (61)	48 (58)
Yes	86 (38)	53 (37)	33 (40)
Mutation status			
KRAS			
No	113 (50)	100 (70)	13 (16)
Yes	37 (16)	37 (26)	0 (0)
Unknown	75 (33)	5 (4)	70 (84)
EGFR			
No	147 (65)	101 (71)	46 (55)
Yes	20 (9)	20 (14)	0 (0)
Unknown	58 (26)	21 (15)	37 (45)
Follow-up			
Mean RFS	3.84 years	4.21 years	3.08 years
Mean OS	6.13 years	6.93 years	4.19 years

Note: NSCLC, non-small cell lung cancer; RFS, recurrence-free survival; OS, overall survival.
*American Joint Committee on Cancer classification.
†Adjuvant therapy status was unknown in 3 patients with adenocarcinoma and 2 patients with squamous cell carcinoma.

Supplementary Table 8. Characteristics of multiplex immunofluorescence panels using Opal 7 and coumarin fluorophores (Akoya Biosciences).

Panel	Antibody	Clone	Vendor	Catalog#	AR	Antibody dilution	F	F dilution
1	CK	AE1/AE3	DAKO	M351501-2	PH6	1:50	620	1:100
	CD3	Polyclonal	DAKO	A045201-2	PH6	1:100	690	1:100
	CD8	C8/144B	Thermo Scientific	MS-457s	PH6	1:25	540	1:100
	PD-1	[EPR4877(2)]	ABCAM	AB137132	PH9	1:3000	650	1:200
	PD-L1	E1L3N	Cell Signaling	13684S	PH6	1:1000	570	1:100
	CD68	PG-M1	DAKO	M087601-2	PH6	1:25	520	1:100
2	CK	AE1/AE3	DAKO	M351501-2	PH6	1:50	620	1:100
	CD3	Polyclonal	DAKO	A045201-2	PH9	1:100	690	1:100
	CD8	C8/144B	Thermo Scientific	MS-457s	PH6	1:25	540	1:100
	CD45RO	UCHL1	Leica Biosystems	PA0146	PH6	RTU	520	1:100
	GZB	11F1	Leica Biosystems	PA0291	PH9	RTU	570	1:100
	FOXP3	D2W8E	Cell Signaling	98377S	PH6	1:50	650	1:200
3	CK	AE1/AE3	DAKO	M351501-2	PH6	1:25	Coumarin	1:100
	CD3	Polyclonal	DAKO	A045201-2	PH6	1:200	650	1:300
	PD-L1	E1L3N	Cell Signaling	13684S	PH6	1:3000	570	1:100
	B7-H3	D9M2L	Cell Signaling	14058S	PH9	1:200	540	1:100
	B7-H4	D1M8I	Cell Signaling	14572S	PH9	1:500	480	1:100
	IDO-1	SP260	ABCAM	AB228468	PH6	1:800	620	1:100
	VISTA	D1L2G	Cell Signaling	64953S	PH9	1:50	520	1:100
4	CK	AE1/AE3	DAKO	M351501-2	PH9	1:25	Coumarin	1:100
	CD3	polyclonal	DAKO	A045201-2	PH6	1:200	690	1:100
	ICOS	D1K2T	Cell Signaling	89601S	PH6	1:200	520	1:100
	LAG3	D2G4O	Cell Signaling	15372S	PH6	1:200	650	1:200
	OX40	ACT-3	Affimetrix (eBioscience)	14-1347-82	PH6	1:10	570	1:100
	TIM3	D5D5R	Cell Signaling	45208S	PH6	1:100	540	1:100
	CD20	L26	DAKO	M075501-2	PH9	1:400	620	1:100
5	CK	AE1/AE3	DAKO	M351501-2	PH6	1:50	Coumarin	1:150
	Arg-1	D4E3M	Cell Signaling	93668S	PH6	1:250	620	1:100
	CD11b	EPR1344	ABCAM	AB133357	PH6	1:6000	650	1:100
	CD14	SP192	ABCAM	AB183322	PH6	1:300	480	1:100
	CD33	[PWS44 (M)]	Leica Biosystems	PA0555	PH9	1:50	520	1:100
	CD66b	G10F5	BioLegend	305102	PH6	1:100	540	1:150
	CD68	PG-M1	DAKO	M087601-2	PH6	1:50	570	1:100

Note: AR, antigen retrieval; CK, cytokeratin; GZB, granzyme B; F, fluorophore.
