# Supplementary material for: Immune cellular patterns of distribution affect outcomes of patients with non-small cell lung cancer
Source: Nat Commun. 2023 Apr 25;14:2364. doi: 10.1038/s41467-023-37905-y (PMC10130161; doi:10.1038/s41467-023-37905-y)
Supplement: Supplementary file 2 — Reporting Summary [file 41467_2023_37905_MOESM2_ESM.pdf]

nature portfolio

Corresponding author(s): Edwin Roger Parra

Last updated by author(s): Apr 4, 2023

Reporting Summary

Nature Portfolio wishes to improve the reproducibility of the work that we publish. This form provides structure for consistency and transparency in reporting. For further information on Nature Portfolio policies, see our Editorial Policies and the Editorial Policy Checklist.

Statistics

For all statistical analyses, confirm that the following items are present in the figure legend, table legend, main text, or Methods section.

n/a	Confirmed	
☐	☒	The exact sample size (*n*) for each experimental group/condition, given as a discrete number and unit of measurement
☐	☒	A statement on whether measurements were taken from distinct samples or whether the same sample was measured repeatedly
☐	☒	The statistical test(s) used AND whether they are one- or two-sided<br>*Only common tests should be described solely by name; describe more complex techniques in the Methods section.*
☐	☒	A description of all covariates tested
☐	☒	A description of any assumptions or corrections, such as tests of normality and adjustment for multiple comparisons
☐	☒	A full description of the statistical parameters including central tendency (e.g. means) or other basic estimates (e.g. regression coefficient) AND variation (e.g. standard deviation) or associated estimates of uncertainty (e.g. confidence intervals)
☐	☒	For null hypothesis testing, the test statistic (e.g. F, t, r) with confidence intervals, effect sizes, degrees of freedom and P value noted<br>*Give P values as exact values whenever suitable.*
☒	☐	For Bayesian analysis, information on the choice of priors and Markov chain Monte Carlo settings
☒	☐	For hierarchical and complex designs, identification of the appropriate level for tests and full reporting of outcomes
☒	☐	Estimates of effect sizes (e.g. Cohen's *d*, Pearson's *r*), indicating how they were calculated

Our web collection on statistics for biologists contains articles on many of the points above.

Software and code

Policy information about availability of computer code

Data collection	The stained slides were scanned using the multispectral microscope Vectra Polaris 1.0.13 imaging system (Akoya Biosciences), and the image analysis was performed using the InForm 2.4.0 digital image analysis software (Akoya Biosciences). Finally, the data were consolidated using R studio 3.5.3 (Phenopter 0.2.2 packet; https://rdrr.io/github/akoyabio/phenoptrReports/f/, Akoya Biosciences).<br>The dimensional reduction was applied using uniform manifold approximation and projection (UMAP) plotted using Python v. 3.8.9. (https://github.com/lmcinnes/umap).<br><br>For spatial pattern distributions was used the cross-G function curve with the theoretical Poisson curve (https://research.csiro.au/software/r-workshop-notes) and the nearest neighbor function (Phenopter 0.2.2 packet; https://rdrr.io/github/akoyabio/phenoptrReports/f/, Akoya Biosciences).
Data analysis	All analyses and data visualization were performed in R 3.6.0 and 3.6.1 (released April 2019; https://www.r-project.org), R studio 3.5.3 (Phenopter 0.2.2 packet; https://rdrr.io/github/akoyabio/phenoptrReports/f/, Akoya Biosciences), Python v.3.8.9, and GraphPad Prism v.9.0.0.

For manuscripts utilizing custom algorithms or software that are central to the research but not yet described in published literature, software must be made available to editors and reviewers. We strongly encourage code deposition in a community repository (e.g. GitHub). See the Nature Portfolio guidelines for submitting code & software for further information.

Data

Policy information about availability of data

All manuscripts must include a data availability statement. This statement should provide the following information, where applicable:

- Accession codes, unique identifiers, or web links for publicly available datasets
- A description of any restrictions on data availability
- For clinical datasets or third party data, please ensure that the statement adheres to our policy

The authors declare that the data supporting the findings of this study are available within the manuscript and its supplementary information files. The data is provided as a source data file. Other relevant de-identified data images related to the current study are available in the repository, https://bitbucket.org/chuymtz/tma3/src/master/ from the corresponding author (E.R.P) upon academic request and will require the researcher to sign a data access agreement with the University of Texas MD Anderson Cancer Center after approval.

Human research participants

Policy information about studies involving human research participants and Sex and Gender in Research.

Reporting on sex and gender	However, clinicopathologic information, including demographic data, age, sex, tobacco history, smoking status, tumor size, tumor stage, adjuvant treatment, and mutational tumor status (KRAS or EGFR), was collected from medical records as shown in the Supplementary Table 7. Sex was not used for any comparison because the patients were not intervening to see the effectiveness of any treatment.
Population characteristics	Clinicopathologic information was obtained, and the cohort included 225 non-small cell lung cancer patients with a median age of 66 years old, 107 females, and 118 males. Tabaco's history has 18 no-smokers and 207 smokers. The median size of the tumor is 3.5cm. Tumor status T1=70, T2=90, T3=40, T4=25; Nodal status N0=148, N1=46, N2=31 and Stage, I=102, II=60, III=63. Adjuvant therapy, No=134, and Yes=86. Mutation status KRAS=37, no or unknown=188; EGFR=20, no or unknown=205. The mean recurrence-free survival was 3.84 years, and the mean overall survival was 6.13 years.
Recruitment	Available tissue specimens were obtained from the Lung Cancer Specialized Program of Research Excellence tissue bank at University of Texas MD Anderson Cancer Center from 225 specimens from patients with stage I-III primary non-small cell lung cancer and placed in TMAs. The samples were collected from patients who had not received neoadjuvant therapy and were evaluated and underwent surgical resection at The University of Texas MD Anderson Cancer Center between 1997 and 2012.
Ethics oversight	The Institutional Review Board (P50CA70907). he University of Texas, MD Anderson Cancer Center approved the study.

Note that full information on the approval of the study protocol must also be provided in the manuscript.

Field-specific reporting

Please select the one below that is the best fit for your research. If you are not sure, read the appropriate sections before making your selection.

☒ Life sciences ☐ Behavioural & social sciences ☐ Ecological, evolutionary & environmental sciences

For a reference copy of the document with all sections, see nature.com/documents/nr-reporting-summary-flat.pdf

Life sciences study design

All studies must disclose on these points even when the disclosure is negative.

Sample size	No sample size calculation was perforated. The maximum available tissue specimens were obtained from the Tissue Biospecimen, and Pathology Resource underwent surgical resection at The University of Texas MD Anderson Cancer Center between 1997 and 2012. The patients had not received neoadjuvant therapy. Two hundred twenty-five patients with stage I-III primary NSCLC, 142 adenocarcinomas (ADCs), and 83 squamous cell carcinomas (SCCs) were obtained and paced in TMAs.
Data exclusions	For cellular spatial analyses, all the populations with very low cell densities equal to or less than two cells/mm2 were excluded to avoid biasing in the analysis, mainly because the analysis was performed in a small area of the TMA's cores.
Replication	Several times the panels were stained in tissue controls obtaining the same pater of staining and results showing the reproducibility of the assay. However, a small cohort of lung cancer was studied previously with similar multiplex immunofluorescence panels and similar methodology for the cellular spatial distribution analysis and obtained similar results ([https://pubmed.ncbi.nlm.nih.gov/33875760/), the experiments and quantifications related to the presented results of the actual manuscript were conducted once.
Randomization	Randomization of the cohort was not essential in the present study because it studied the overall cellular distribution of the tumor-associated immune cells in non-small cell lung cancer tissue samples. In addition, the study was not designed to compare the effectiveness of any treatment on the patients.

Blinding

Blinding for data collection or analysis was irrelevant to this study because the study was not designed to be any intervention or treatment for the patients.

Reporting for specific materials, systems and methods

We require information from authors about some types of materials, experimental systems and methods used in many studies. Here, indicate whether each material, system or method listed is relevant to your study. If you are not sure if a list item applies to your research, read the appropriate section before selecting a response.

Materials & experimental systems

n/a	Involved in the study
☐	☒ Antibodies
☒	☐ Eukaryotic cell lines
☒	☐ Palaeontology and archaeology
☒	☐ Animals and other organisms
☒	☐ Clinical data
☒	☐ Dual use research of concern

Methods

n/a	Involved in the study
☒	☐ ChIP-seq
☒	☐ Flow cytometry
☒	☐ MRI-based neuroimaging

Antibodies

Antibodies used

Multiplex Immunofluorescence staining: antibodies against (Panel 1): cytokeratin (clone AE1/AE3, cat# M351501-2, dilution 1:50, Dako, Santa Clara, CA), CD3 (polyclonal, cat#A045201-2, dilution 1:100, Dako), CD8 (clone C8/144B, cat# MS-457s, dilution 1:25, Thermo Fisher Scientific, Waltham, MA), PD-1 (clone EPR4877-2, cat# AB137132, dilution 1:3000, Abcam, Cambridge, MA), PD-L1 (clone E1L3N, cat#13684S, dilution 1:1000, Cell Signaling, Danvers, MA), and CD68 (clone PG-M1, cat# M087601-2, dilution 1:25, Dako), (Panel 2): cytokeratin (clone AE1/AE3, cat# M351501-2, dilution 1:50, Dako), CD3 (polyclonal, cat# A045201-2, dilution 1:100, Dako), CD8 (clone C8/144B, cat# MS-457s, dilution 1:25, Thermo Fisher Scientific), CD45RO (clone UCHL1, cat# PA0146, Cell Signaling), Granzyme B (clone 11F1, cat# PA0291, Cell Signaling), and FOXP3 (clone D2W8E, cat#98377S, dilution 1:50, Cell Signaling), (Panel 3): cytokeratin (clone AE1/AE3, cat# M351501-2, dilution 1:25, Dako), CD3 (polyclonal, cat#A045201-2, dilution 1:200, Dako), PD-L1 (clone E1L3N, cat#13684S, dilution 1:3000, Cell Signaling), B7-H3 (clone D9M2L, cat#14058S, dilution 1:200,Cell Signaling), B7-H4 (D1M8I, cat#14572S, dilution 1:500, Cell Signaling), IDO-1 (clone SP260, cat# AB228468, dilution 1:800, Abcam), and VISTA (clone D1L2G, cat#64953S, dilution 1:50, Cell Signaling), (Panel 4): cytokeratin (clone AE1/AE3, cat# M351501-2, dilution 1:25, Dako), CD3 (polyclonal, cat#A045201-2, dilution 1:200, Dako), ICOS (clone D1K2T, cat#89601S, dilution 1:200, Cell Signaling), LAG3 (clone D2G4O, cat#15372S, dilution 1:200, Cell Signaling), OX40 (ACT-3, cat#14-1347-82, dilution 1:10, Affimetrix, eBioscience), TIM3 (clone D5D5R, cat#45208S, dilution 1:100, Cell Signaling), and CD20 (clone L26, cat# M075501-2, dilution 1:400, Dako) and, (Panel 5): cytokeratin (clone AE1/AE3, cat# M351501-2, dilution 1:50, Dako), Arg-1 (clone D4E3M, cat#93668S, dilution 1:250, Cell Signaling), CD11b (clone EPR1344, cat# AB133357, dilution 1:600, Abcam), CD14 (clone SP192, cat# AB183322, dilution 1:300, Abcam), CD33 [clone (PWS44 (M), cat# PA0555, dilution 1:50, Leica Biosystems], CD66b (clone G10F5, cat#305102, dilution 1:100, BioLegend), and CD68 (clone PG-M1, cat# M087601-2, dilution 1:50, Dako). All markers were stained in sequence using their respective fluorophore contained in the Opal 7 IHCkit (cat#NEL797001KT; Akoya Biosciences, Waltham, MA) for the panels with 6 antibodies, and coumarin fluorophore (cat# NEL703001KT) was added in the panels with 7 antibodies.

Validation

Multiplex immunofluorescence antibodies were previously validated as reported in https://pubmed.ncbi.nlm.nih.gov/33875760/
